# Supplementary material for: Identification of Freezing-Responsive microRNAs and Their Targets in Chinese Jujube by Small RNA and Degradome Sequencing
Source: Int J Mol Sci. 2026 Sep 4;27(17):7912. doi: 10.3390/ijms27177912 (PMC13566890; doi:10.3390/ijms27177912)
Supplement: Supplementary file 1 [file ijms-27-07912-s001.zip › Supplementary Material S2/novel_miR_74.pdf]

Provisional ID	:	novel_miR_74
Score total	:	71764.6
Score for star read(s)	:	3.9
Score for read counts	:	71758
Score for mfe	:	1
Score for randfold	:	1.6
Score for cons. seed	:	
Total read count	:	140762
Mature read count	:	140253
Loop read count	:	1
Star read count	:	508

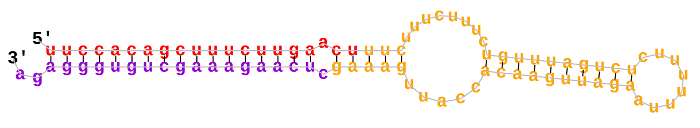

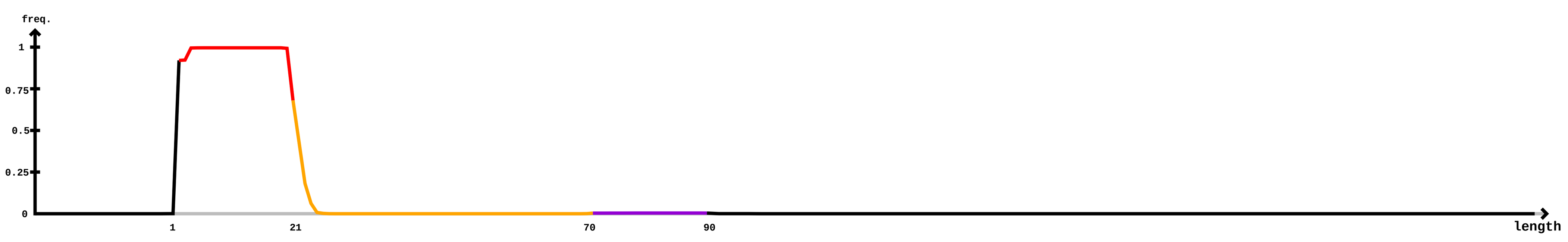


```
                       Mature                                                     Star
5'- uuugcaaguccuggucaugcuuuuccacagcuuucuugaacuuucuuucuuucuguuuagucucuuuuuuaagauugaacaccauugaaagcucaagaaagcugugggagaauauggcaaauuugggauuuuaauuuuuuuauuuuuguuuuuauuuuuuauuuuuccuucaacuacucuucagcugaugaugauguugcacacagaaagaaagaaagaaagaaagaaagagagaucucagaagcuaaau  -3'  obs
    uuugcaaguccuggucaugcuuuuccacagcuuucuugaacuuucuuucuuucuguuuagucucuuuuuuaagauugaacaccauugaaagcucaagaaagcugugggagaauauggcaaauuugggauuuuaauuuuuuuauuuuuguuuuuauuuuuuauuuuuccuucaacuacucuucagcugaugaugauguugcacacagaaagaaagaaagaaagaaagaaagagagaucucagaagcuaaau       exp
    ............(((((.((((((((((((((((((.(((((........((((((((((........))))))))))......))))).)))))))))))))))))).))))))...((((((((((..((((((.(((((.(((((.((((((.........((((...((.(((.....))).)).))))......)))))).))))).))))).))))))))))))))))........  reads  mm  sample
    ....................uuuuccacagcuuucuugaac.................................................................................................................................................................................................................  6  0  JCK-2
    ....................uuuuccacagcuuucuugaac.................................................................................................................................................................................................................  3  0  LT-3
    ....................uuuuccacagcuuucuugaac.................................................................................................................................................................................................................  3  0  LT-1
    ....................uuuuccacagcuuucuugaac.................................................................................................................................................................................................................  3  0  JT-3
    ....................uuuuccacagcuuucuugaac.................................................................................................................................................................................................................  2  0  JT-2
    ....................uuuuccacagcuuucuugaac.................................................................................................................................................................................................................  3  0  LCK-3
    ....................uuuuccacagcuuucuugaac.................................................................................................................................................................................................................  4  0  JCK-1
    ....................uuuuccacagcuuucuugaac.................................................................................................................................................................................................................  7  0  JCK-3
    ....................uuuuccacagcuuucuugaacu................................................................................................................................................................................................................  4  0  JCK-2
    ....................uuuuccacagcuuucuugaacu................................................................................................................................................................................................................  1  0  JCK-3
    ....................uNuuccacagcuuucuugaacu................................................................................................................................................................................................................  1  1  JCK-3
    .....................uuuccacagcuuucuuga...................................................................................................................................................................................................................  2  0  JCK-1
    .....................Auuccacagcuuucuugaacu................................................................................................................................................................................................................  2  1  JCK-2
    .....................Auuccacagcuuucuugaacu................................................................................................................................................................................................................  1  1  JCK-3
    .....................uuuccacagcuuucuugaacu................................................................................................................................................................................................................  1  0  JCK-3
    .....................Guuccacagcuuucuugaacu................................................................................................................................................................................................................  2  1  LT-3
    .....................uuuccacagcuuucuugaacu................................................................................................................................................................................................................  1  0  LT-1
    .....................uuuccacagcuuucuugaacu................................................................................................................................................................................................................  1  0  JCK-2
    .....................uuuccacagcuuucuugaacu................................................................................................................................................................................................................  2  0  LCK-3
    .....................Cuuccacagcuuucuugaacuu...............................................................................................................................................................................................................  1  1  LT-2
    .....................Cuuccacagcuuucuugaacuu...............................................................................................................................................................................................................  3  1  JCK-2
    .....................Cuuccacagcuuucuugaacuu...............................................................................................................................................................................................................  2  1  JCK-1
    .....................Cuuccacagcuuucuugaacuu...............................................................................................................................................................................................................  3  1  LT-1
    .....................Cuuccacagcuuucuugaacuu...............................................................................................................................................................................................................  3  1  LT-3
    .....................Cuuccacagcuuucuugaacuuu..............................................................................................................................................................................................................  2  1  JCK-1
    .....................Cuuccacagcuuucuugaacuuu..............................................................................................................................................................................................................  1  1  LT-3
    .....................Cuuccacagcuuucuugaacuuu..............................................................................................................................................................................................................  1  1  LT-1
    .....................Cuuccacagcuuucuugaacuuu..............................................................................................................................................................................................................  3  1  LCK-3
    ......................uuccacagcuuucuugCa..................................................................................................................................................................................................................  1  1  LT-2
    ......................uuccacagcuuucuugaC..................................................................................................................................................................................................................  4  1  LT-2
    ......................uuccacagcuuucuugaC..................................................................................................................................................................................................................  1  1  JCK-3
    ......................uuccacagcuuucuuUaa..................................................................................................................................................................................................................  1  1  LT-1
    ......................uuccacagcuuucuugGa..................................................................................................................................................................................................................  1  1  LCK-2
```

Mature **Star**

uuugcaaguccuggucaugcuu**uuccacagcuuucuugaacu**uucuuucuuucuguuuagucucuuuuuuaagauugaacaccauugaaag**cucaagaaagcugugggaga**auauggcaaauuugggauuuuaauuuuuuuauuuuuguuuuuauuuuuuauuuuuccuucaacuacucuucagcugaugaugauguugcacacagaaagaaagaaagaaagaaagaaagagagaucucagaagcuaaau

......................uuccacagcuuucuugaa	15	0	JCK-2
......................uuccacagcuuucuugUa	4	1	LT-3
......................uuccacagcuuucuugUa	1	1	LT-1
......................uuccacagcuuucuugaU	1	1	JCK-2
......................uuccacagcuuucuugaU	11	1	LT-1
......................uuccacagcuuucuugUa	19	1	LCK-3
......................uuccacagcuuucuugaa	53	0	LCK-2
......................uuccacagcuuucuugaa	15	0	JT-3
......................uuccacagcuuucuugUa	2	1	JCK-3
......................uuccacagcuuucuugaa	43	0	LT-2
......................uuccacagcuuucuugaa	52	0	LT-3
......................uuccacagcuuucuugaa	9	0	JCK-3
......................uuccacagcuuucuugaa	5	0	JT-2
......................uuccacagcuuucuGgaa	1	1	LCK-1
......................uuccaNagcuuucuugaa	1	1	LCK-1
......................uuccacagcuuucuugaa	31	0	LT-1
......................uuccacagcuuucuugaU	4	1	LCK-3
......................uuccacagcuuucuugUa	5	1	JT-1
......................uuccacagcuuucuugaa	16	0	JCK-1
......................uuccacagcuuucuugaC	1	1	LT-3
......................uuccacagcuuucuugaU	3	1	LT-2
......................uuccacagcuuucuugaU	14	1	LT-3
......................uuccacagcuuucuugUa	15	1	LT-2
......................uuccacagcuuucuugaU	1	1	JCK-1
......................uuccacagcuuucuugaa	46	0	LCK-1
......................uuccacagcuuucuugaa	43	0	LCK-3
......................Cuccacagcuuucuugaac	3	1	LCK-3
......................uuccacagcuuucCugaac	1	1	JCK-3
......................uuccacagcuuucuugCac	11	1	LT-3
......................uucUacagcuuucuugaac	4	1	JCK-2
......................uuccacagcuuucuugCac	1	1	LCK-1
......................uuNcacagcuuucuugaac	1	1	LCK-1
......................uNccacagcuuucuugaac	1	1	JCK-1
......................uNccacagcuuucuugaac	2	1	JCK-3
......................uuccacCgcuuucuugaac	1	1	JT-2
......................uCccacagcuuucuugaac	1	1	JCK-2
......................uuccacagcuuCcuugaac	2	1	JCK-3
......................uuUcacagcuuucuugaac	2	1	JCK-2
......................uuccacagcuuucuugaGc	1	1	LT-3
......................uuccacagcuuucuugaaU	53	1	JCK-2
......................uuccacagUuuucuugaac	4	1	LCK-3
......................uNccacagcuuucuugaac	1	1	LCK-2
......................uuccCcagcuuucuugaac	33	1	LT-2
......................uuccCcagcuuucuugaac	1	1	JCK-1
......................uuccCcagcuuucuugaac	12	1	LT-1
......................uuccacagcuuucCugaac	2	1	LCK-3
......................uuccacagcuuucuugaac	5694	0	LT-2
......................uuccacagcuuucuugCac	5	1	JCK-2
......................uuUcacagcuuucuugaac	6	1	JCK-1
......................uuccacagcuuucuugaUc	10	1	LCK-3
......................uCccacagcuuucuugaac	1	1	JCK-1
......................uuccacagcuuucuugaaU	64	1	LT-2
......................uuccacagcuuCcuugaac	3	1	LCK-3
......................uuccaUagcuuucuugaac	1	1	LT-2
......................uucUacagcuuucuugaac	2	1	JCK-1
......................uuccCcagcuuucuugaac	16	1	LT-3
......................uuccaAagcuuucuugaac	3	1	LT-3
......................uuccaAagcuuucuugaac	1	1	LCK-3
......................uuccacagcCuucuugaac	1	1	LT-2
......................uuccacagcCuucuugaac	1	1	JCK-1
......................uuccacagcuuucuugCac	4	1	LT-1
......................uuccacagAuuucuugaac	1	1	LT-2
......................uuccacagcuuucuuUaac	1	1	JT-1
......................uuccCcagcuuucuugaac	4	1	JCK-2
......................uuccacagcuuucuugaac	1739	0	LCK-2
......................uuccaUagcuuucuugaac	1	1	JCK-2
......................uuccaAagcuuucuugaac	1	1	JCK-2
......................uCccacagcuuucuugaac	1	1	LCK-1
......................uuccacagcuuCcuugaac	3	1	JCK-1
......................uuccacagcuuucuugaUc	3	1	LT-2

Mature **Star**

uuugcaaguccuggucaugcuu**uuccacagcuuucuugaacu**uucuuucuuucuguuuagucucuuuuuuaagauugaacaccauugaaag**cucaagaaagcugugggaga**auauggcaaauuugggauuuuaauuuuuuuauuuuuguuuuuauuuuuuauuuuuccuucaacuacucuucagcugaugaugauguugcacacagaaagaaagaaagaaagaaagaaagagagaucucagaagcuaaau

uuccacagcuuucuugaaU	37	1	LT-1
uuccacagcuuCcuugaac	3	1	JCK-2
uuccacagcuuucuugaUc	2	1	JCK-1
Cuccacagcuuucuugaac	4	1	LT-1
uuccacagcuuuUuugaac	6	1	LT-2
uuccacagUuuucuugaac	5	1	LT-1
uuccacagcuuucuugaac	6061	0	LT-1
uuccacGgcuuucuugaac	2	1	JCK-2
uuccacagcuuucuugUac	1	1	LT-3
uuccacagcuCucuugaac	1	1	LT-1
uuccCcagcuuucuugaac	2	1	JCK-3
uuNcacagcuuucuugaac	14	1	LT-2
uuccacagcuuucCugaac	2	1	LT-1
uuccacaAcuuucuugaac	1	1	LT-3
uuccacagcuuucuugaaU	24	1	JCK-1
uuccacagcuGucuugaac	1	1	LT-1
uuccacagcuuuUuugaac	2	1	JCK-3
uuUcacagcuuucuugaac	1	1	LT-1
uuccacCgcuuucuugaac	12	1	LT-1
uuccacagcuuucuugCac	1	1	JCK-3
uuccacagcuuucuCgaac	1	1	LT-1
uuccacagcuuucGugaac	3	1	LCK-1
uuccacagcuuucuugaCc	1	1	JCK-3
uuccacagcuuucuugaGc	1	1	LCK-3
uuccacaAcuuucuugaac	2	1	LT-2
uuccacagcuuucuugaaU	103	1	LCK-3
uuccacagcuuucuugaCc	7	1	JCK-2
uuccacagcuuucuugaac	8638	0	LT-3
uuccacCgcuuucuugaac	10	1	LT-2
uuccacagUuuucuugaac	3	1	LT-2
uucUacagcuuucuugaac	1	1	JCK-3
uuccacagcuuCcuugaac	2	1	LT-1
uCccacagcuuucuugaac	2	1	LT-3
uNccacagcuuucuugaac	3	1	JT-1
uuccacagcuuucuGgaac	1	1	JCK-2
uuccacagcuuucuugCac	3	1	LCK-3
uuccGcagcuuucuugaac	1	1	LT-1
uuccacagcuuucuugCac	3	1	JT-1
uuccacagcuuucuugaac	2667	0	JCK-3
uuccacagcuuucuugaUc	2	1	LT-3
uuccacagcuuuNuugaac	1	1	LCK-1
uuccacCgcuuucuugaac	2	1	JCK-1
uuccacagcuuucuugaac	1676	0	LCK-1
uuccCcagcuuucuugaac	1	1	JT-3
uuccacagcuuucuugCac	2	1	JCK-1
uuccacagcuuucuugaaU	26	1	LCK-1
uuccacagcuuucuuAaac	4	1	LT-2
uuccacagcuCucuugaac	2	1	JCK-2
Cuccacagcuuucuugaac	1	1	JCK-3
uuccacagcuuucuugaaU	24	1	LCK-2
uuccacCgcuuucuugaac	11	1	LCK-3
uuccacagcuuucuugaCc	8	1	LCK-3
uuccacCgcuuucuugaac	9	1	JCK-2
uuccCcagcuuucuugaac	2	1	LCK-1
uuccacagcuGucuugaac	3	1	JCK-1
uuccacaUcuuucuugaac	2	1	JCK-2
uuccacagcuuucuugaCc	10	1	LT-3
uuccacaAcuuucuugaac	2	1	LCK-3
uuccacagcuuucuugaCc	1	1	JT-1
uuAcacagcuuucuugaac	1	1	JCK-1
uuccacagcuuucuGgaac	2	1	LCK-1
uuccacagcuuucuugaac	1423	0	JT-1
uuccacagcuuucNugaac	6	1	LCK-1
uuccacagcuuucuugaCc	2	1	LCK-2
uuUcacagcuuucuugaac	1	1	JCK-3
uuccacCgcuuucuugaac	2	1	LCK-2
uuccacagcuuucuuAaac	1	1	JCK-1
uuccaUagcuuucuugaac	2	1	LT-3
uNccacagcuuucuugaac	5	1	JCK-2
Cuccacagcuuucuugaac	1	1	LT-2

Mature **Star**

uuugcaaguccuggucaugcuuuuccacagcuuucuugaacuuucuuucuuucuguuuagucucuuuuuuaagauugaacaccauugaaagcucaagaaagcugugggagaauauggcaaauuugggauuuuaauuuuuuuauuuuuguuuuuauuuuuuauuuuuccuucaacuacucuucagcugaugaugauguugcacacagaaagaaagaaagaaagaaagaaagagagaucucagaagcuaaau			
......................uuccacagcuuuUuugaac	4	1	JCK-1
......................uuccacagcuuucuugaCc	5	1	LT-1
......................uuccacagcCuucuugaac	1	1	JCK-2
......................uuccaNagcuuucuugaac	3	1	LCK-1
......................uuccacaAcuuucuugaac	5	1	LT-1
......................uucNacagcuuucuugaac	1	1	JCK-1
......................uuccacagcuuucuugaac	3571	0	JCK-2
......................uuccacagcuuuUuugaac	1	1	LCK-2
......................uuccacagcuuuUuugaac	8	1	JCK-2
......................uuccacagcCuucuugaac	5	1	LT-3
......................uuccCcagcuuucuugaac	2	1	JT-1
......................uuccacagcuuuAuugaac	1	1	LT-2
......................uuccacagcuuNcuugaac	2	1	JCK-1
......................Cuccacagcuuucuugaac	3	1	LT-3
......................uuccacagcuuucuugaCc	13	1	LT-2
......................uuccacagcuuucCugaac	1	1	LT-2
......................uuccacagcCuucuugaac	7	1	LT-1
......................uuccacaAcuuucuugaac	1	1	JCK-2
......................uuccCcagcuuucuugaac	13	1	LCK-3
......................uuccacagcuuucuugUac	3	1	LCK-3
......................Cuccacagcuuucuugaac	2	1	JT-1
......................uuGcacagcuuucuugaac	3	1	LT-2
......................uuccacagcuuucuugGac	1	1	JCK-3
......................uuccacCgcuuucuugaac	2	1	JCK-3
......................uuUcacagcuuucuugaac	1	1	LT-3
......................uuccCcagcuuucuugaac	1	1	LCK-2
......................uucAacagcuuucuugaac	1	1	LT-2
......................uuccacagcuuuAuugaac	2	1	LCK-3
......................uuccacagUuuucuugaac	1	1	LT-3
......................uuccacagcuuucuugaac	411	0	JT-2
......................uuccaAagcuuucuugaac	1	1	LT-2
......................uuccacagcuuucuugaGc	1	1	JCK-1
......................uuccacagcuuuAuugaac	2	1	LT-1
......................uuccacagcuuucuugCac	5	1	LT-2
......................uuccacagcuuucuugaaU	98	1	LT-3
......................uuccacaAcuuucuugaac	1	1	JCK-3
......................uuccacagcuuucuugaac	8415	0	LCK-3
......................uuccacagcuuuUuugaac	3	1	LT-1
......................uuccacagcuuucuNgaac	4	1	LCK-1
......................uuccacagcuuucuugCac	1	1	LCK-2
......................uuccacagcuuucCugaac	3	1	JCK-2
......................uuccacagcuuucuugaUc	3	1	JT-1
......................uuAcacagcuuucuugaac	1	1	LT-3
......................uuccacagcuuucuugaac	542	0	JT-3
......................uuccacagcuuucuugaCc	1	1	LCK-1
......................uuccacagcuuucuugaac	2180	0	JCK-1
......................uuccacCgcuuucuugaac	16	1	LT-3
......................uuccacagcuuucuugaaU	28	1	JCK-3
......................uuccaAagcuuucuugaac	1	1	JCK-1
......................uuccacCgcuuucuugaac	2	1	JT-1
......................uuccacagcuuucuugaaNu	1	1	LT-1
......................uuccacagcuuucuugaacA	20	1	JT-1
......................uuccacagcuuucuCgaacu	1	1	LT-2
......................uuccacagcuuucuugaCcu	8	1	LT-3
......................uuccacagcuuucuCgaacu	1	1	LT-3
......................uuUcacagcuuucuugaacu	1	1	JCK-3
......................uuccacagcuuucuugaCcu	1	1	LT-1
......................uuccacagcuuucuuAaacu	3	1	LCK-3
......................uuccaNagcuuucuugaacu	3	1	LCK-1
......................uuccCcagcuuucuugaacu	8	1	LT-3
......................uuccacagcuuucCugaacu	1	1	JT-1
......................uuccacagcuuucuCgaacu	1	1	JCK-1
......................uuccacagcuuucNugaacu	5	1	LCK-1
......................uuccacagcuuucuugaaAu	2	1	LT-3
......................uuccacagcuuucuugaaUu	154	1	LT-2
......................uuccacagcuuucuugaacC	2	1	LCK-1
......................uuccacagcuuucuugaacu	2055	0	JT-1
......................uuccacaUcuuucuugaacu	1	1	JCK-1
......................uCccacagcuuucuugaacu	2	1	JCK-2
......................uuccCcagcuuucuugaacu	1	1	JCK-2

```
                      Mature                                                                       Star
uuugcaaguccuggucaugcuuuuccacagcuuucuugaacuuucuuucuuucuguuuagucucuuuuuuaagauugaacaccauugaaagcucaagaaagcugugggagaauauggcaaauuugggauuuuaauuuuuuuauuuuuguuuuuauuuuuuauuuuuccuucaacuacucuucagcugaugaugauguugcacacagaaagaaagaaagaaagaaagaaagagagaucucagaagcuaaau
......................uuccacagcuuucuugaaAu................................................................................................................................................................................   1   1   JCK-3
......................uuccacagcuuuAuugaacu................................................................................................................................................................................   2   1   JCK-3
......................uuUcacagcuuucuugaacu................................................................................................................................................................................   4   1   JCK-2
......................Cuccacagcuuucuugaacu................................................................................................................................................................................   2   1   LCK-1
......................uuccacagcuGucuugaacu................................................................................................................................................................................   1   1   LT-2
......................uuccacagcuuucGugaacu................................................................................................................................................................................   1   1   LCK-2
......................uuccacagcuuucuugaCcu................................................................................................................................................................................   3   1   JCK-2
......................uuccacagcuCucuugaacu................................................................................................................................................................................   2   1   LT-2
......................uuccacagcuuCcuugaacu................................................................................................................................................................................   1   1   JCK-2
......................uuccacagcuuucCugaacu................................................................................................................................................................................   1   1   LT-1
......................uuccaAagcuuucuugaacu................................................................................................................................................................................   2   1   JCK-1
......................uuccacagcuuucuugaacC................................................................................................................................................................................   18   1   LT-3
......................uuccacagcuuucuugaacA................................................................................................................................................................................   57   1   JCK-2
......................uuccaAagcuuucuugaacu................................................................................................................................................................................   3   1   LT-1
......................uuccacagcuuucuugaacA................................................................................................................................................................................   196   1   LCK-3
......................uuccaAagcuuucuugaacu................................................................................................................................................................................   1   1   JCK-3
......................uuccacCgcuuucuugaacu................................................................................................................................................................................   2   1   LCK-1
......................uuccacagcCuucuugaacu................................................................................................................................................................................   1   1   JT-3
......................uuccGcagcuuucuugaacu................................................................................................................................................................................   1   1   LCK-3
......................uuccacagcuuNcuugaacu................................................................................................................................................................................   3   1   JT-1
......................uuccacagcuuucCugaacu................................................................................................................................................................................   1   1   LT-3
......................uuccacagcuuuAuugaacu................................................................................................................................................................................   1   1   LT-2
......................uuccacagcCuucuugaacu................................................................................................................................................................................   3   1   JCK-2
......................uuccacagcuuucuugaGcu................................................................................................................................................................................   3   1   JCK-1
......................uuccacagcuuucuuUaacu................................................................................................................................................................................   1   1   LT-2
......................uuccacagcCuucuugaacu................................................................................................................................................................................   7   1   LT-2
......................uuccacagcuuucuugaacA................................................................................................................................................................................   87   1   LT-1
......................uuccacagcuuucuugaacA................................................................................................................................................................................   137   1   LT-2
......................uuccacagcuuucuugaacA................................................................................................................................................................................   2   1   LCK-1
......................uuccacagUuuucuugaacu................................................................................................................................................................................   1   1   JT-1
......................uuccacagcuuucGugaacu................................................................................................................................................................................   1   1   LCK-1
......................uuccacagcuuucuugaCcu................................................................................................................................................................................   3   1   JT-1
......................uuccacagcuuuUuugaacu................................................................................................................................................................................   2   1   LT-1
......................Cuccacagcuuucuugaacu................................................................................................................................................................................   1   1   LT-3
......................uuccacagcuuucuGgaacu................................................................................................................................................................................   1   1   LT-3
......................uuccacagcuuucuugaaUu................................................................................................................................................................................   192   1   LCK-3
......................uuccacagcuuGcuugaacu................................................................................................................................................................................   1   1   JCK-1
......................uuccacagcuuucuugaNcu................................................................................................................................................................................   1   1   LCK-1
......................uuccacagcuuucuugaacG................................................................................................................................................................................   8   1   LT-2
......................Cuccacagcuuucuugaacu................................................................................................................................................................................   1   1   JCK-2
......................uuccacagcuuucuugCacu................................................................................................................................................................................   10   1   LCK-3
......................uuccacagcuuucuugaacA................................................................................................................................................................................   11   1   JT-3
......................uuccacagcuuucuugaacA................................................................................................................................................................................   43   1   JCK-3
......................uuccacagcuuucuNgaacu................................................................................................................................................................................   7   1   LCK-1
......................uuccaAagcuuucuugaacu................................................................................................................................................................................   1   1   LCK-1
......................uuccacagcuuucuugaacC................................................................................................................................................................................   9   1   LT-1
......................uuccacagcuuucuugaacC................................................................................................................................................................................   41   1   LCK-3
......................uNccacagcuuucuugaacu................................................................................................................................................................................   4   1   JCK-1
......................Cuccacagcuuucuugaacu................................................................................................................................................................................   2   1   LT-1
......................uuccacagcuuucuugaacG................................................................................................................................................................................   6   1   LT-3
......................uuccacagcuuucuugaacu................................................................................................................................................................................   544   0   JT-2
......................uNccacagcuuucuugaacu................................................................................................................................................................................   1   1   JT-1
......................uuccacagcuuucuugaGcu................................................................................................................................................................................   2   1   LCK-3
......................uuccacagcuuucuugaacC................................................................................................................................................................................   5   1   JCK-3
......................uucUacagcuuucuugaacu................................................................................................................................................................................   3   1   LT-2
......................uuNcacagcuuucuugaacu................................................................................................................................................................................   33   1   LT-2
......................uuccacagcuuucuugaacA................................................................................................................................................................................   3   1   JT-2
......................uuccaAagcuuucuugaacu................................................................................................................................................................................   1   1   JCK-2
......................uuccacCgcuuucuugaacu................................................................................................................................................................................   3   1   JCK-3
......................uuccCcagcuuucuugaacu................................................................................................................................................................................   4   1   JCK-1
......................Cuccacagcuuucuugaacu................................................................................................................................................................................   2   1   JCK-3
......................uuccacagcuCucuugaacu................................................................................................................................................................................   1   1   LCK-3
......................uuccacagcuuucuugaacu................................................................................................................................................................................   731   0   JT-3
......................uuccacagcuuucuugaUcu................................................................................................................................................................................   2   1   LT-1
......................uuccacagcuuucuugaacG................................................................................................................................................................................   2   1   LT-1
......................uuccacagcuuucuugaCcu................................................................................................................................................................................   11   1   LCK-3
......................uuccacagcuuucuugaacC................................................................................................................................................................................   7   1   JCK-1
......................uNccacagcuuucuugaacu................................................................................................................................................................................   2   1   LCK-1
......................uuccacCgcuuucuugaacu................................................................................................................................................................................   4   1   LT-1
......................uuccacagcuuucuGgaacu................................................................................................................................................................................   1   1   JT-1
```

```
                     Mature                                                                Star
uugcaaguccuggucaugcuuuuccacagcuuucuugaacuuucuuucuuucuguuuagucucuuuuuuaagauugaacaccauugaaagcucaagaaagcugugggagaauauggcaaauuugggauuuuaauuuuuuuauuuuuguuuuuauuuuuuauuuuuccuucaacuacucuucagcugaugaugauguugcacacagaaagaaagaaagaaagaaagaaagagagaucucagaagcuaaau
.....................uuccacagcuuucuugaacC...............................................................................................................................................................................   2      1    JCK-2
.....................uuccacCgcuuucuugaacu...............................................................................................................................................................................   3      1    LCK-2
.....................uuccacagcuCucuugaacu...............................................................................................................................................................................   3      1    LT-1
.....................uucNacagcuuucuugaacu...............................................................................................................................................................................   2      1    JCK-1
.....................uNccacagcuuucuugaacu...............................................................................................................................................................................   1      1    JCK-2
.....................uuccacCgcuuucuugaacu...............................................................................................................................................................................   8      1    LT-3
.....................uuccacagcuuucuugCacu...............................................................................................................................................................................   2      1    LCK-1
.....................uuccacagcuuuAuugaacu...............................................................................................................................................................................   4      1    LCK-3
.....................uuccCcagcuuucuugaacu...............................................................................................................................................................................   2      1    JT-3
.....................uuccacagcuuucuugaCcu...............................................................................................................................................................................   1      1    JCK-3
.....................uuccCcagcuuucuugaacu...............................................................................................................................................................................   20     1    LT-2
.....................uuccacagcuuucuugaacG...............................................................................................................................................................................   45     1    LCK-3
.....................uuccacagcuuucuugaacC...............................................................................................................................................................................   3      1    JT-3
.....................uuccacagcCuucuugaacu...............................................................................................................................................................................   5      1    LT-1
.....................uuccacaAcuuucuugaacu...............................................................................................................................................................................   1      1    LCK-2
.....................uuccacagUuuucuugaacu...............................................................................................................................................................................   1      1    JCK-2
.....................uuccUcagcuuucuugaacu...............................................................................................................................................................................   1      1    JCK-2
.....................uuccCcagcuuucuugaacu...............................................................................................................................................................................   3      1    LT-1
.....................uuccacagcuuucuugCacu...............................................................................................................................................................................   10     1    LT-1
.....................uuccacagcuuucuugaacu...............................................................................................................................................................................   10755  0    LCK-3
.....................uCccacagcuuucuugaacu...............................................................................................................................................................................   1      1    LT-1
.....................uCccacagcuuucuugaacu...............................................................................................................................................................................   2      1    JCK-1
.....................uuccacagcuuucuugaaAu...............................................................................................................................................................................   6      1    LCK-3
.....................uuccacagcuuuUuugaacu...............................................................................................................................................................................   1      1    LT-3
.....................uuccacagcuuuAuugaacu...............................................................................................................................................................................   2      1    LT-3
.....................uuccacGgcuuucuugaacu...............................................................................................................................................................................   2      1    JT-3
.....................uuccacagcuuucuugaaUu...............................................................................................................................................................................   132    1    LT-1
.....................uuccacagcuuucuugaacu...............................................................................................................................................................................   2125   0    JCK-1
.....................uuccacagcuuucuugaacC...............................................................................................................................................................................   39     1    LT-2
.....................uuccacCgcuuucuugaacu...............................................................................................................................................................................   3      1    JCK-1
.....................uuccacCgcuuucuugaacu...............................................................................................................................................................................   6      1    JCK-2
.....................uuccacagcuuucuugaacN...............................................................................................................................................................................   2      1    JCK-1
.....................uuccacagcuuucuugaacu...............................................................................................................................................................................   2316   0    LCK-1
.....................uuccacagcuuucuugaacu...............................................................................................................................................................................   3389   0    JCK-2
.....................uuccacagcuuNcuugaacu...............................................................................................................................................................................   3      1    JCK-1
.....................uucUacagcuuucuugaacu...............................................................................................................................................................................   1      1    JCK-1
.....................uuccaAagcuuucuugaacu...............................................................................................................................................................................   7      1    LCK-3
.....................uuccacagcuuucuugaCcu...............................................................................................................................................................................   25     1    LT-2
.....................uuccacagcuuucuugaaUu...............................................................................................................................................................................   45     1    JT-2
.....................uuccacagcuuucCugaacu...............................................................................................................................................................................   2      1    LCK-3
.....................uuccacagcuuucuugaaUu...............................................................................................................................................................................   24     1    LCK-1
.....................uuccacagcuuCcuugaacu...............................................................................................................................................................................   1      1    LCK-3
.....................uuccacagcuuucuugaacC...............................................................................................................................................................................   8      1    JT-1
.....................uuccacagcuuucuugaacA...............................................................................................................................................................................   172    1    LT-3
.....................uuccacagcuuucuugCacu...............................................................................................................................................................................   3      1    JCK-2
.....................uuccacagcuuucuuAaacu...............................................................................................................................................................................   1      1    JCK-1
.....................uNccacagcuuucuugaacu...............................................................................................................................................................................   2      1    JCK-3
.....................uuccacagcuuucuugaaUu...............................................................................................................................................................................   50     1    JT-3
.....................uuccacagcuuucuugCacu...............................................................................................................................................................................   6      1    LT-3
.....................uuccacagcuuucuugaacu...............................................................................................................................................................................   2326   0    LCK-2
.....................uuccacagcuuucuugaaUu...............................................................................................................................................................................   72     1    JCK-1
.....................uuccacCgcuuucuugaacu...............................................................................................................................................................................   9      1    LCK-3
.....................uuccacagcCuucuugaacu...............................................................................................................................................................................   1      1    JCK-3
.....................uuccacagcuuucuugaacC...............................................................................................................................................................................   3      1    JT-2
.....................uuccUcagcuuucuugaacu...............................................................................................................................................................................   1      1    JCK-1
.....................uuccacagcuuucuugaacG...............................................................................................................................................................................   1      1    JT-1
.....................uuccacagcuuucuGgaacu...............................................................................................................................................................................   4      1    LCK-1
.....................uuccacaAcuuucuugaacu...............................................................................................................................................................................   4      1    LCK-3
.....................uCccacagcuuucuugaacu...............................................................................................................................................................................   1      1    LCK-3
.....................uuccacagcuuucuugaCcu...............................................................................................................................................................................   1      1    LCK-2
.....................uuccacagcuuucuuAaacu...............................................................................................................................................................................   1      1    LT-2
.....................uuccacagcuuucuugaaAu...............................................................................................................................................................................   6      1    LT-2
.....................uuccacagcuuucuugaaAu...............................................................................................................................................................................   1      1    JCK-1
.....................uuccacagcCuucuugaacu...............................................................................................................................................................................   1      1    JCK-1
.....................uuccacagcuuucuugaaAu...............................................................................................................................................................................   2      1    LT-1
.....................uuccacaAcuuucuugaacu...............................................................................................................................................................................   1      1    LT-2
.....................uuccacagcuuucuugCacu...............................................................................................................................................................................   3      1    JCK-1
.....................uuccacagcuuCcuugaacu...............................................................................................................................................................................   2      1    LT-3
.....................uuccacagcuuucuugCacu...............................................................................................................................................................................   10     1    LT-2
.....................uuccacaAcuuucuugaacu...............................................................................................................................................................................   1      1    JT-1
```

Mature · **Star**

uuugcaaguccuggucaugcuuuuccacagcuuucuugaacuuucuuucuuucuguuuagucucuuuuuuaagauugaacaccauugaaagcucaagaaagcugugggagaauauggcaaauuugggauuuuaauuuuuuuauuuuuguuuuuauuuuuuauuuuuccuucaacuacucuucagcugaugaugauguugcacacagaaagaaagaaagaaagaaagaaagagagaucucagaagcuaaau

uuccacagcuuucuugGacu	1	1	LT-3
uCccacagcuuucuugaacu	1	1	LT-3
uuccacagcuCucuugaacu	1	1	JT-1
uuccacagcuuucuugaCcu	1	1	JT-2
uuccacagcuuucuugaaUu	35	1	LCK-2
uucUacagcuuucuugaacu	3	1	LCK-3
uuccacaAcuuucuugaacu	1	1	LT-1
uuccacagcuuucuugaNcu	2	1	JCK-3
uuccacagcuuucuugaacu	6887	0	LT-2
uuccacagUuuucuugaacu	1	1	LCK-3
uuccacagcuuucuuCaacu	1	1	JCK-1
uuccacagcuuucuugaaUu	52	1	JCK-3
uuccacagcuuucCugaacu	1	1	JCK-2
uuccacagUuuucuugaacu	1	1	LT-1
uuccacagcuuucuugaaUu	111	1	JCK-2
uuccacagcuuucuugaaUu	172	1	LT-3
uuccacagcuuucuugGacu	1	1	LT-2
uuccCcagcuuucuugaacu	1	1	LCK-1
uuGcacagcuuucuugaacu	5	1	LT-2
uuccacCgcuuucuugaacu	1	1	JT-1
uuccCcagcuuucuugaacu	8	1	LCK-3
uuccacagcuuucuugaCcu	3	1	LCK-1
uuccacagcuuuNuugaacu	1	1	LCK-1
uuccacagcuuucuugaacA	48	1	JCK-1
uuccacagcuuucuugaaUu	18	1	JT-1
uuccacagcuuucuugaacu	7087	0	LT-3
uuccacCgcuuucuugaacu	13	1	LT-2
uuccacagcuuucuugaCcu	3	1	JCK-1
uuccacagcuuucuugaacu	2309	0	JCK-3
uuccacagcuuucuugGacu	8	1	LCK-3
Cuccacagcuuucuugaacu	4	1	LCK-3
uuccacagcuuucuugaUcu	2	1	LT-2
uuccacagcuuucuugaacu	4415	0	LT-1
Cuccacagcuuucuugaacu	1	1	LT-2
uuccaUagcuuucuugaacu	3	1	JCK-2
uCccacagcuuucuugaacu	3	1	JCK-3
uuccacagcuGucuugaacu	1	1	JCK-1
uuccacagcuuucuugaacG	2	1	JCK-2
uuccacagcuuNcuugaacu	1	1	JT-2
uuccacaAcuuucuugaacu	1	1	LT-3
uuccacagcCuucuugaacu	1	1	LT-3
uuccacagcuuucuugCacu	2	1	JCK-3
uuNcacagcuuucuugaacu	2	1	LCK-3
uuccCcagcuuucuugaacu	2	1	JT-1
uuccacGgcuuucuugaacu	1	1	LCK-3
uuccacagcuuucuugCacu	2	1	LCK-2
uuccacagcuuCcuugaacu	2	1	LT-2
uuccacagcCuucuugaacu	1	1	JT-2
uuccaAagcuuucuugaacu	3	1	LT-3
uuccacagcuuucuugaacuC	33	1	LT-1
uuccacagcuuucuugaCcuu	3	1	JCK-1
uuccaAagcuuucuugaacuu	5	1	JCK-2
uuccacagcuuucuugaaAuu	3	1	LCK-3
uuUcacagcuuucuugaacuu	2	1	JT-2
uucGacagcuuucuugaacuu	1	1	JCK-1
uuccacagcuuucuugaacuC	6	1	LCK-1
uuccacagcuuucuugaaUuu	11	1	JT-3
uuccacCgcuuucuugaacuu	1	1	JT-1
uuccacagcuuucuugCacuu	2	1	LT-3
uuccacagcuuucuugaacuA	5	1	JT-3
uuccacagcuuucuugaaUuu	29	1	LCK-1
uucUacagcuuucuugaacuu	4	1	JCK-2
Cuccacagcuuucuugaacuu	1	1	LT-3
uuccacaAcuuucuugaacuu	1	1	JCK-3
uuccacagcuuucuugaacuu	2427	0	LCK-3
uuccacagcuuucuugaacuC	54	1	LT-3
uuccacagcuuucuugaacuu	502	0	JT-1
uuccacagcuuucuugaacuA	97	1	JT-1
uuccacagcuuucuugaacCu	1	1	LT-2
uuccacagcuuucuugCacuu	2	1	LT-2

```
                      Mature                                                                 Star
uuugcaaguccuggucaugcuuuuccacagcuuucuugaacuuucuuucuuucuguuuagucucuuuuuuaagauugaacaccauugaaagcucaagaaagcugugggagaauauggcaaauuugggauuuuaauuuuuuuauuuuuguuuuuauuuuuuauuuuuccuucaacuacucuucagcugaugaugauguugcacacagaaagaaagaaagaaagaaagaaagagagaucucagaagcuaaau
......................uNccacagcuuucuugaacuu...............................................................................................................................................................................................................  6     1  JCK-3
......................uuccacagcuuucuuAaacuu...............................................................................................................................................................................................................  1     1  JCK-1
......................uuccacCgcuuucuugaacuu...............................................................................................................................................................................................................  1     1  LCK-2
......................uuccacagcuuucuugaacNu...............................................................................................................................................................................................................  2     1  JCK-1
......................uuccacagcuuucuugaCcuu...............................................................................................................................................................................................................  3     1  LCK-3
......................uuccacagcuuucuugaaUuu...............................................................................................................................................................................................................  50    1  JCK-1
......................uuccacaAcuuucuugaacuu...............................................................................................................................................................................................................  1     1  LT-1
......................uuccacagcuuucuugaacuC...............................................................................................................................................................................................................  53    1  LCK-3
......................uNccacagcuuucuugaacuu...............................................................................................................................................................................................................  4     1  JCK-2
......................uuccCcagcuuucuugaacuu...............................................................................................................................................................................................................  5     1  LT-2
......................uucUacagcuuucuugaacuu...............................................................................................................................................................................................................  1     1  JCK-3
......................uuccacagcuuucuugaaUuu...............................................................................................................................................................................................................  135   1  LT-3
......................uuccacagcCuucuugaacuu...............................................................................................................................................................................................................  2     1  LT-1
......................uuccacagcuuucuugaacuC...............................................................................................................................................................................................................  9     1  LCK-2
......................uuccCcagcuuucuugaacuu...............................................................................................................................................................................................................  6     1  JCK-2
......................uuccacagcuuucuugaacuu...............................................................................................................................................................................................................  1431  0  LT-2
......................uuccacagcuuucuugaacuC...............................................................................................................................................................................................................  64    1  JCK-2
......................uuccacGgcuuucuugaacuu...............................................................................................................................................................................................................  1     1  JCK-1
......................uuccacagcuuucuugaacAu...............................................................................................................................................................................................................  6     1  LT-3
......................uuccacagcuuucuugaacuA...............................................................................................................................................................................................................  125   1  JCK-2
......................uuccacagcuuucuugaGcuu...............................................................................................................................................................................................................  4     1  JCK-1
......................uCccacagcuuucuugaacuu...............................................................................................................................................................................................................  1     1  JCK-2
......................uuccacagcuuucuugaacuA...............................................................................................................................................................................................................  148   1  LT-3
......................uuccacagcuuucuugaaUuu...............................................................................................................................................................................................................  88    1  JCK-2
......................uuccaAagcuuucuugaacuu...............................................................................................................................................................................................................  3     1  JCK-3
......................uuccacagcCuucuugaacuu...............................................................................................................................................................................................................  1     1  JCK-3
......................uuccacagcuuuUuugaacuu...............................................................................................................................................................................................................  1     1  LT-1
......................uucNacagcuuucuugaacuu...............................................................................................................................................................................................................  1     1  JCK-3
......................uuccaUagcuuucuugaacuu...............................................................................................................................................................................................................  1     1  LT-3
......................uuccacagcuuucuugaacuu...............................................................................................................................................................................................................  145   0  JT-3
......................uCccacagcuuucuugaacuu...............................................................................................................................................................................................................  1     1  LCK-2
......................uuccacagcuuucuugaacuA...............................................................................................................................................................................................................  2     1  JT-2
......................uuccacagcuuucuugaCcuu...............................................................................................................................................................................................................  2     1  JT-1
......................uuccacagcuuucuugaacuu...............................................................................................................................................................................................................  3888  0  JCK-2
......................uuUcacagcuuucuugaacuu...............................................................................................................................................................................................................  1     1  JT-3
......................uuUcacagcuuucuugaacuu...............................................................................................................................................................................................................  2     1  JCK-3
......................uuccacagcuuuAuugaacuu...............................................................................................................................................................................................................  1     1  LT-3
......................uuccacagcuuucuugaacuu...............................................................................................................................................................................................................  191   0  LCK-2
......................uuccacagcuuucuugaacAu...............................................................................................................................................................................................................  8     1  LCK-3
......................uuccacagcuuucuugaaUuu...............................................................................................................................................................................................................  86    1  LT-1
......................uuccacagcuuuUuugaacuu...............................................................................................................................................................................................................  1     1  JCK-2
......................uuccaAagcuuucuugaacuu...............................................................................................................................................................................................................  2     1  JCK-1
......................uuccacagcuuucuugCacuu...............................................................................................................................................................................................................  1     1  JT-1
......................uuccacagcuuucCugaacuu...............................................................................................................................................................................................................  1     1  LT-3
......................uuccacagcuuucuugaaUuu...............................................................................................................................................................................................................  3     1  JT-2
......................uuccacagcuuucuuAaacuu...............................................................................................................................................................................................................  1     1  LT-1
......................uuccacagcuuucuugaacuC...............................................................................................................................................................................................................  44    1  LT-2
......................uuccacagcuuucuugaacuC...............................................................................................................................................................................................................  53    1  JCK-1
......................uuccacagNuuucuugaacuu...............................................................................................................................................................................................................  1     1  JCK-3
......................uuccacagcuuucuugaacAu...............................................................................................................................................................................................................  1     1  LT-1
......................uuccacagcuuucCugaacuu...............................................................................................................................................................................................................  1     1  JCK-2
......................uuccacagcuuucuugaaAuu...............................................................................................................................................................................................................  5     1  LT-2
......................uuccacagcuuucuugaacAu...............................................................................................................................................................................................................  3     1  JCK-3
......................uuccacagcuuucuugaacuA...............................................................................................................................................................................................................  67    1  JCK-3
......................uucNacagcuuucuugaacuu...............................................................................................................................................................................................................  2     1  JCK-1
......................uuccacagcuuucuugCacuu...............................................................................................................................................................................................................  1     1  LCK-3
......................uuccacagcuuucuugaaUuu...............................................................................................................................................................................................................  101   1  LT-2
......................uuccacagcuuuAuugaacuu...............................................................................................................................................................................................................  1     1  LCK-3
......................uuccacagcuuucuugaacuA...............................................................................................................................................................................................................  66    1  LT-1
......................uuccCcagcuuucuugaacuu...............................................................................................................................................................................................................  2     1  JCK-1
......................uuccacagcuuucuugaacuu...............................................................................................................................................................................................................  2955  0  JCK-3
......................uCccacagcuuucuugaacuu...............................................................................................................................................................................................................  1     1  LT-2
......................uuccacagcuuucuugaacAu...............................................................................................................................................................................................................  2     1  JCK-1
......................uuccacagcuuucuugaacuu...............................................................................................................................................................................................................  1769  0  LT-3
......................uuccacGgcuuucuugaacuu...............................................................................................................................................................................................................  7     1  JT-2
......................uuccacGgcuuucuugaacuu...............................................................................................................................................................................................................  11    1  JT-3
......................uuccacagcuuucuugaCcuu...............................................................................................................................................................................................................  2     1  JCK-3
......................uuccacagcuuucuugaacuA...............................................................................................................................................................................................................  75    1  JCK-1
......................uuccCcagcuuucuugaacuu...............................................................................................................................................................................................................  3     1  LT-3
......................uuNcacagcuuucuugaacuu...............................................................................................................................................................................................................  4     1  LT-2
```

Mature | Star

Sequence	Count	Mismatch	Library
uugcaaguccuggucaugcuuuuccacagcuuucuugaacuuucuuucuuucuguuuagucucuuuuuuaagauugaacaccauugaaagcucaagaaagcugugggagaauauggcaaauuugggauuuuaauuuuuuuauuuuuguuuuuauuuuuuauuuuuccuucaacuacucuucagcugaugaugauguugcacacagaaagaaagaaagaaagaaagaaagagagaucucagaagcuaaau			
.....................uuccacagcuuucuuAaacuu...	2	1	LCK-3
.....................uuccaUagcuuucuugaacuu...	3	1	JCK-3
.....................uuccacagcuuucuugaacuu...	954	0	LT-1
.....................uuccacagcuuucuugaacuu...	157	0	LCK-1
.....................uuccacagcuuucuugaacAu...	5	1	JCK-2
.....................uuccacagcuuCcuugaacuu...	1	1	LT-2
.....................uNccacagcuuucuugaacuu...	1	1	JT-1
.....................uuccacagcuuucuugCacuu...	2	1	JCK-3
.....................uuccacagcuuucuugaacuC...	18	1	JT-1
.....................uuccacaAcuuucuugaacuu...	1	1	LCK-3
.....................uuccacGgcuuucuugaacuu...	1	1	JCK-2
.....................uuccacagcuuucuugaacuC...	68	1	JCK-3
.....................uuccCcagcuuucuugaacuu...	1	1	LCK-3
.....................uuccacagcuuucuugaacGu...	1	1	JCK-2
.....................uuccacCgcuuucuugaacuu...	3	1	JCK-2
.....................uCccacagcuuucuugaacuu...	1	1	LCK-3
.....................uuccacagcuuucuugaaUuu...	112	1	LCK-3
.....................uuccacagcuuucuugaacGu...	1	1	LT-3
.....................uuccacagcuuucuugaacuA...	134	1	LT-2
.....................uuccacagcuuucuugCacuu...	1	1	LT-1
.....................uuccacagcuuucuugaacuA...	20	1	LCK-1
.....................uuccacaAcuuucuugaacuu...	1	1	LT-2
.....................uuUcacagcuuucuugaacuu...	6	1	JCK-2
.....................uuccaUagcuuucuugaacuu...	1	1	JCK-1
.....................Cuccacagcuuucuugaacuu...	3	1	JCK-3
.....................uNccacagcuuucuugaacuu...	1	1	LT-2
.....................uuccacagcuuucCugaacuu...	1	1	JCK-1
.....................uuccacagcuuucuugaaUuu...	48	1	LCK-2
.....................uuccacagcuuuAuugaacuu...	1	1	LT-2
.....................uuccacagcuuucuugaacuN...	4	1	JCK-1
.....................uucNacagcuuucuugaacuu...	1	1	JT-1
.....................uuccacagcuuucuugaacuu...	97	0	JT-2
.....................uuccacagcuuucuugaacGu...	1	1	LT-1
.....................uuccacagcuuucuugaCcuu...	5	1	JCK-2
.....................uuccacagcuuucuugaaUuu...	61	1	JCK-3
.....................uuccaNagcuuucuugaacuu...	2	1	LCK-1
.....................uuccacCgcuuucuugaacuu...	5	1	JCK-1
.....................uuccacagcuuucuugaacAu...	6	1	LT-2
.....................uuccacagcuuucuugaacuu...	2349	0	JCK-1
.....................uuccacagcuuucuCgaacuu...	5	1	LT-2
.....................uuccacagcuuucuugaaAuu...	1	1	LT-1
.....................uuccacCgcuuucuugaacuu...	3	1	LCK-3
.....................uuccacagcuuucuugaacuA...	240	1	LCK-3
.....................uuccacagcuuucuuAaacuu...	1	1	LCK-2
.....................uuccacagcuuucuugaacuA...	23	1	LCK-2
.....................uuccacagcuuucuugCacuu...	2	1	JCK-1
.....................uuccacagcuCucuugaacuu...	1	1	JT-2
.....................uNccacagcuuucuugaacuu...	1	1	JCK-1
.....................uuccacagcuuucuugaCcuu...	4	1	LT-2
.....................uuccacCgcuuucuugaacuu...	3	1	JCK-3
.....................uuccacagcuuNcuugaacuu...	3	1	JCK-1
.....................uuccacagcuuucuugaCcuu...	1	1	LT-3
.....................uuccacCgcuuucuugaacuu...	3	1	LT-3
.....................uuUcacagcuuucuugaacuu...	1	1	JCK-1
.....................uuccaAagcuuucuugaacuu...	1	1	LT-2
.....................uuccacagAuuucuugaacuu...	1	1	JCK-1
.....................uuccCcagcuuucuugaacuu...	2	1	JCK-3
.....................uuccacagcuuuAuugaacuu...	3	1	JCK-3
.....................uuccacagcuuucuugCacuu...	5	1	JCK-2
.....................uuccacagcuuGcuugaacuuu...	2	1	JCK-1
.....................uuccacagcuuucuugaacuAu...	13	1	LT-2
.....................uuccacagcuuucuugaacuuC...	73	1	LT-1
.....................uuccacagcuuucuugaacCuu...	1	1	LT-3
.....................uuccacagcuuucuugaacAuu...	29	1	LT-3
.....................uuccacagcuuucuugaacuuG...	2	1	JCK-2
.....................uuUcacagcuuucuugaacuuu...	7	1	LT-2
.....................uuccacagcuuucuugaaAuuu...	2	1	JT-3
.....................Cuccacagcuuucuugaacuuu...	1	1	LT-3
.....................uuccacagcuuucuugaaAuuu...	4	1	JCK-1
.....................uuccacagcuuucuugaacAuu...	14	1	JCK-1

Mature: uuccacagcuuucuugaacu; Star: cucaagaaagcugugggaga			
uuugcaaguccuggucaugcuuuuccacagcuuucuugaacuuucuuucuuucuguuuagucucuuuuuuaagauugaacaccauugaaagcucaagaaagcugugggagaauauggcaaauuugggauuuuaauuuuuuuauuuuuguuuuuauuuuuuauuuuuccuucaacuacucuucagcugaugaugauguugcacacagaaagaaagaaagaaagaaagaaagagagaucucagaagcuaaau			
uuccacagcuuuAuugaacuuu	4	1	LT-2
uuccacagcuuuAuugaacuuu	1	1	LT-1
uuccacagcuuucuugaacuCu	2	1	JCK-1
uuccacagcuuucuugaaAuuu	2	1	LT-3
uuccacagcuuucuugaacCuu	3	1	JCK-3
uuGcacagcuuucuugaacuuu	2	1	LT-2
uucNacagcuuucuugaacuuu	1	1	JCK-1
uuccacagcuuucuugaacuuC	118	1	LT-3
uuccacagcuuucuugaacuuC	55	1	JCK-3
uuccacagcuuucuugaacuuA	77	1	JCK-3
uuccaNagcuuucuugaacuuu	1	1	LCK-1
uuUcacagcuuucuugaacuuu	1	1	JCK-2
uuccacagcuuuAuugaacuuu	2	1	JCK-1
uuccacagcuuucuugaaAuuu	3	1	JCK-2
uuccacagcuuucuugaacuCu	1	1	LT-2
uuccacagcuuucuugaacuuA	10	1	JT-2
uuccaAagcuuucuugaacuuu	1	1	JCK-1
uuccacagcuuucCugaacuuu	1	1	JCK-2
uuccCcagcuuucuugaacuuu	4	1	LT-3
uCccacagcuuucuugaacuuu	1	1	LT-1
uuccacagcuuucuugaacuAu	16	1	JCK-2
uuccacCgcuuucuugaacuuu	2	1	JCK-2
uuccacagcuuucuugaacuuC	8	1	JT-2
uuccacagcuuucuugaacuuC	12	1	JT-3
uuccacagcuuucuugaCcuuu	2	1	LT-1
uuccacagcuuucuugaacuuA	62	1	LT-1
uuccacagcuuucuugaacuuA	17	1	JT-3
uuccacagcuuucuugaacuuu	64	0	JT-3
uuccacagcCuucuugaacuuu	2	1	JCK-1
uuccacagcuuucuugaacAuu	12	1	JCK-3
uuccCcagcuuucuugaacuuu	4	1	LCK-3
uNccacagcuuucuugaacuuu	1	1	JCK-1
uuccacagcuuucuugaacuAu	6	1	JCK-3
uuccacagcuuucuugaaUuuu	3	1	JT-2
uuccacagcuuucuugaacuuu	70	0	JT-2
uuccacagcuuCcuugaacuuu	1	1	LT-2
uuccacagcuuucuugaCcuuu	2	1	LT-3
uuccacagcuuucuugaacuuG	7	1	LT-2
uuccacagcuGucuugaacuuu	1	1	LT-2
uuccacagcuuucuugaacuuA	121	1	LT-3
uuccacagcuuucuugaaAuuu	2	1	JCK-3
uuccacaAcuuucuugaacuuu	1	1	JT-3
uGccacagcuuucuugaacuuu	1	1	LCK-3
uuccacagcuuucuugCacuuu	2	1	LT-2
uuccacagcuuucuugaacuuC	253	1	LCK-3
uuccacagcuuucuugaacuuA	31	1	LCK-2
uuccacagcuuucuugaacuCu	1	1	LT-1
uuccacagcuuucuugaCcuuu	1	1	LCK-2
uuccacagcuuucuugaacuuu	1068	0	LT-1
uuccacagcuuucuugaacuuC	114	1	LT-2
uuccCcagcuuucuugaacuuu	1	1	LT-1
uuccacagcuuucuugCacuuu	2	1	LT-1
uuccacagcuuucuugaacuuC	65	1	JCK-1
uuccacagcuuucuugaacuuu	1512	0	LT-3
uuccacagcuuucuugaacGuu	8	1	JCK-2
uuccCcagcuuucuugaacuuu	1	1	JCK-1
uuccacCgcuuucuugaacuuu	2	1	LT-3
uuccacagcuuucuugCacuuu	1	1	JCK-2
uuccacagcuuucuugaaUuuu	36	1	JCK-3
uuccacagcuuucuugaacuCu	3	1	LT-3
uuccacagcuuucuugaacuAu	17	1	LCK-3
uuccacagcuuucuugaacuNu	1	1	JT-2
uuccacagcuuucuugaaUuuu	22	1	LCK-1
uuccacagcuuucuugaacuCu	5	1	JCK-3
uuccacagcuuucuugaacuuN	1	1	LT-2
uuccacCgcuuucuugaacuuu	1	1	LCK-3
uuccacagcuuucuugaacuuA	50	1	JCK-1
uuccacagcuuucuugaacAuu	36	1	JCK-2
uuccCcagcuuucuugaacuuu	2	1	JCK-2
uuccaAagcuuucuugaacuuu	1	1	JCK-2

Mature Star

uuugcaaguccuggucaugcuuuuccacagcuuucuugaacuuucuuucuuucuguuuagucucuuuuuuaagauugaacaccauugaaagcucaagaaagcugugggagaauauggcaaauuugggauuuuaauuuuuuuauuuuuguuuuuauuuuuuauuuuuccuucaacuacucuucagcugaugaugauguugcacacagaaagaaagaaagaaagaaagaaagagagaucucagaagcuaaau			
......................uuccacagcuuNcuugaacuuu	3	1	JCK-1
......................uuccacagcuuucuugaacCuu	1	1	JCK-1
......................uuccacagcuuucuugaaUuuu	13	1	LCK-2
......................uuccacagcuuucuugaaUuuu	27	1	JCK-1
......................uuccacaAcuuucuugaacuuu	2	1	LT-2
......................uuccacagcuuucuugaacAuu	6	1	JT-2
......................uuccacCgcuuucuugaacuuu	1	1	JCK-1
......................uuccacagcCuucuugaacuuu	2	1	JCK-2
......................uuccacagcuuucuugaacuuN	1	1	JCK-1
......................uuccacagcuuucuugaacuuG	3	1	LT-3
......................uuccacagcuuucuugaacuuC	89	1	JCK-2
......................uuccacagcuuucuugaaUuuu	63	1	JCK-2
......................uuccacagcuuucuugCacuuu	1	1	JT-3
......................uuccGcagcuuucuugaacuuu	1	1	LT-2
......................uuccacagcuuucuugaCcuuu	3	1	LT-2
......................uuccacagcuuucuugaaUuuu	32	1	LT-3
......................uuccacagcuGucuugaacuuu	2	1	JCK-1
......................uuccacagcuuucuugaCcuuu	1	1	LCK-3
......................uuccacagcuuucuugCacuuu	1	1	LCK-2
......................uuccacagcuuucuugaacuuG	2	1	LT-1
......................uuccacagcuuucuugaaUuuu	7	1	JT-1
......................uCccacagcuuucuugaacuuu	1	1	JCK-3
......................uuccacagcuuucuugaacuNu	1	1	JCK-1
......................uuccacagcuuucuugaacAuu	16	1	LCK-3
......................uuccacagcuuucuugaacuuA	128	1	JCK-2
......................uuccacagcuuucuugaacuCu	5	1	LCK-3
......................uuccacCgcuuucuugaacuuu	2	1	LCK-2
......................uuccacCgcuuucuugaacuuu	3	1	JCK-3
......................uuccacagcuuucuugaaUuuu	1	1	JT-3
......................uuccacagcuuucuugaacuAu	11	1	LT-1
......................uuccacagcuuucuugaacAuu	4	1	JT-3
......................uuccCcagcuuucuugaacuuu	3	1	LT-2
......................uuUcacagcuuucuugaacuuu	1	1	LT-3
......................uuccacCgcuuucuugaacuuu	1	1	JT-1
......................uuccacagcuuucuugaacCuu	3	1	JCK-2
......................Cuccacagcuuucuugaacuuu	1	1	JCK-3
......................Cuccacagcuuucuugaacuuu	1	1	JCK-1
......................uuccacagcuuucuuUaacuuu	1	1	LT-3
......................uuccacagcuuucuugaacCuu	1	1	LCK-1
......................uuccacagcuuucuugaacuuu	250	0	LCK-1
......................uuccacagcuuucuugCacuuu	1	1	JCK-1
......................uuccacagcuuucuugaacuuA	121	1	LT-2
......................uuccacagcuuucuugCacuuu	1	1	LCK-3
......................uuccaUagcuuucuugaacuuu	1	1	LT-1
......................uuccacagcuCucuugaacuuu	1	1	LT-1
......................uuccacagcuuucuugaacuuC	41	1	JT-1
......................uuccacagcuuucuugaacuuu	1496	0	JCK-2
......................uuccacagcuuucuugCacuuu	1	1	JCK-3
......................uuccacagcuuucuugaacuuu	2105	0	LCK-3
......................uuccacagcCuucuugaacuuu	4	1	JCK-3
......................uuccacagcCuucuugaacuuu	2	1	LT-2
......................uuccacagcuuucuugaaUuuu	37	1	LT-2
......................uuccacagcuuucuugaacuuA	21	1	LCK-1
......................uuccacagcuuucuugaacAuu	26	1	LT-2
......................uuccacagcuuucuugaacuAu	3	1	JCK-1
......................uuccacCgcuuucuugaacuuu	2	1	LT-1
......................uuccacagcuuucuugaacuAu	12	1	LT-3
......................uuccacagcuuucuugaaUuuu	41	1	LT-1
......................uNccacagcuuucuugaacuuu	2	1	LCK-3
......................uuccacagcuuucuugaacuuu	377	0	JT-1
......................uuccCcagcuuucuugaacuuu	1	1	JCK-3
......................uuccacagcuuucuugaacuuA	157	1	LCK-3
......................uNccacagcuuucuugaacuuu	1	1	JCK-3
......................uuccacagcuuucuugaacGuu	3	1	JCK-1
......................uuNcacagcuuucuugaacuuu	4	1	LT-2
......................uuccacagcuuucuugaacuuu	899	0	JCK-1
......................uuUcacagcuuucuugaacuuu	1	1	JCK-1
......................uuccaAagcuuucuugaacuuu	1	1	JCK-3
......................uuccacagcuuNcuugaacuuu	1	1	JT-1
......................uuccacagcuuucuugaacAuu	12	1	LT-1

Mature Star

```
uuugcaaguccuggucaugcuuuuccacagcuuucuugaacuuucuuucuuucuguuuagucucuuuuuuaagauugaacaccauugaaagcucaagaaagcugugggagaauauggcaaauuugggauuuuaauuuuuuuauuuuuguuuuuauuuuuuauuuuuccuucaacuacucuucagcugaugaugauguugcacacagaaagaaagaaagaaagaaagaaagagagaucucagaagcuaaau
```

......................uuccacagcuuucuugaacGuu	1	1	LT-3
......................uuccacCgcuuucuugaacuuu	8	1	LT-2
......................uuccacagcuuucuugaacuuu	1108	0	JCK-3
......................uuccacagcuuucuugaaUuuu	39	1	LCK-3
......................uuccacagcuuucuugaacuuu	246	0	LCK-2
......................uNccacagcuuucuugaacuuu	1	1	JCK-2
......................uuccacagcuuucNugaacuuu	1	1	LCK-1
......................uuccacagcuuucuugaacuCu	6	1	JCK-2
......................uuccacagcuuucuugaCcuuu	2	1	JCK-2
......................uuccacagcuuucuCgaacuuu	3	1	LT-3
......................uuccacagcuuucuugaacuuA	49	1	JT-1
......................Cuccacagcuuucuugaacuuu	1	1	JT-1
......................uucUacagcuuucuugaacuuu	1	1	LCK-3
......................uuccacagcuuucuugaacuuu	1432	0	LT-2
......................uCccacagcuuucuugaacuuu	3	1	LT-3
......................uuccacagcuuucuugaacuuuU	294	1	JCK-1
......................uuccacagcuuucuugaacuuAc	1	1	LT-2
......................uuccacagcuuucuugaacuuuG	4	1	JCK-1
......................uuccacagcuuucuugaacuuuG	13	1	LT-1
......................uuNcacagcuuucuugaacuuuc	1	1	LT-2
......................uuccacagcuuucuugaacuuuU	140	1	LCK-1
......................uuccacagcuuucuugaacuuuU	306	1	LT-1
......................uuccacagcuuucuugaacuuuc	3	0	JT-2
......................uuccacagcuuucuugaacuAuc	7	1	LT-2
......................uuccacagcuuucuugaacuuAc	1	1	LT-3
......................uuccacagcuuucuugaacuuuc	50	0	JCK-2
......................uuccacagcuuucuugaacAuuc	10	1	LCK-3
......................uuccacagcuuucuugaacuuuc	61	0	LT-2
......................uuccacagcuuucuugaaUuuuc	5	1	JCK-3
......................uuccacagcuuucuugaacuuuc	3	0	JT-3
......................uuccacagcuuucuugaacuuuU	324	1	LT-3
......................uuccacagcuuucuugaacAuuc	1	1	JCK-3
......................uuccacagcuuucuugaacuuuc	47	0	LT-3
......................uuccacagcuuucuugaaUuuuc	15	1	LCK-1
......................uuccacagcuuucuugaacuuuA	144	1	JCK-1
......................uuccacagcuuucuugaacuuuG	9	1	LCK-3
......................uuccacCgcuuucuugaacuuuc	1	1	LCK-3
......................uuccacagcuuucuugaacuuuG	11	1	LT-3
......................uuccacagcuuucuugaacuuAc	1	1	JCK-3
......................uuccacagcuuucuugaacuuuU	134	1	LCK-2
......................uuccacagcuuucuugaacuuCc	1	1	LT-1
......................uuccacagcuuucuugaacuuuc	16	0	JT-1
......................uuccacagcuuucuugaacAuuc	2	1	LT-2
......................uuccacagcuuucuugaacuuuG	9	1	LT-2
......................uuccacagcuuucuugaaUuuuc	13	1	LCK-2
......................uuccacagcuuucuugaacuAuc	4	1	LT-1
......................uuccacGgcuuucuugaacuuuc	1	1	LT-2
......................uuccacagcuuucuugaacuuuU	376	1	LT-2
......................uuccacagcuuucuugaacuuuU	490	1	LCK-3
......................uuccacagcuuucuugaacuuuc	97	0	LCK-3
......................uuccacagcuuucuugaacuAuc	1	1	LT-3
......................uuccacagcuuucuugaacuuAc	5	1	JT-3
......................uuccacagcuuucuugaacAuuc	1	1	JCK-1
......................uuccacagcuuucuugaacuuuG	8	1	JCK-2
......................uuccacagcuuucuugaacuuuA	11	1	JT-2
......................uuccacagcuuucuugaaUuuuc	3	1	JCK-2
......................uuccacagcuuucuugaaUuuuc	4	1	LT-1
......................uuccacagcuuucuugaacuuuU	18	1	JT-3
......................uuccacagcuuucuugaaUuuuc	6	1	LCK-3
......................uuccacagcuuucuugaacuuuA	230	1	LT-2
......................uuccacagcuuucuugCacuuuc	1	1	LCK-3
......................uuccacagcuuucuugaacuuuA	301	1	LCK-3
......................uuccacagcuuucuugaacuuuA	166	1	LT-3
......................uuccacagcuuucuugCacuuuc	1	1	LT-2
......................uuccacagcuuucuugaacuuuA	16	1	JT-3
......................uuccacagcuuucuugaacuuuU	492	1	JCK-2
......................uuccacagcuuucuugaacuuuA	119	1	LT-1
......................uuccacagcuuucuugaacAuuc	4	1	LT-1
......................uuccCcagcuuucuugaacuuuc	1	1	LT-2
......................uuccacagcuuucuugaacuuuU	101	1	JT-1

Mature Star

```
uuugcaaguccuggucaugcuuuuccacagcuuucuugaacuuucuuucuuucuguuuagucucuuuuuuaagauugaacaccauugaaagcucaagaaagcugugggagaauauggcaaauuugggauuuuaauuuuuuuauuuuuguuuuuauuuuuuauuuuuccuucaacuacucuucagcugaugaugauguugcacacagaaagaaagaaagaaagaaagaaagagagaucucagaagcuaaau
......................uuccacagcuuucuugaacuuuc     33   0   JCK-1
......................uuccacagcuuucuugaacuuuG     4    1   JCK-3
......................uuccacagcuuucuugaaAuuuc     1    1   LT-3
......................uuccacagcuuucuugaacuuuc     28   0   LT-1
......................uuccacCgcuuucuugaacuuuc     1    1   LT-2
......................uuccacagcuuucuugaacuuuA     48   1   LCK-2
......................uuccacagcuuucuugaacuuuA     40   1   LCK-1
......................uuccacagcuuucuugaacAuuc     3    1   LT-3
......................uuccacagcuuucuugaacuuuA     155  1   JCK-3
......................uuccacagcuuucuugaacuuuU     369  1   JCK-3
......................uuccacagcuuucuugaacuuuA     47   1   JT-1
......................uuccacagcuuucuugaacuuuA     177  1   JCK-2
......................uuccacagcuuucuugaacuuuc     40   0   JCK-3
......................uuccacagcuuucuugaacuuAc     1    1   JT-2
......................uuccacagcuuucuugaacuuuU     9    1   JT-2
......................uuccacagcuuucuugaacuAucu    4    1   JCK-2
......................uuccacagcuuucuugaacuuAcu    2    1   JCK-2
......................uuccacagcuuucuugaacuuucA    1    1   JCK-1
......................uuccacagcuuucuugaacuuucA    6    1   JT-1
......................uuccacagcuuucuugaacuuucA    4    1   JCK-3
......................uuccacagcuuucuugaacuAucu    5    1   LCK-3
......................uuccacagcuuucuugaacuuuAu    7    1   JCK-3
......................uuccacagcuuucuugaacuuuAu    6    1   JCK-2
......................uuccacagcuuucuugaacuuuAu    2    1   JCK-1
......................uuccacagcuuucuugaacuuuAu    5    1   LCK-3
......................uuccacagcuuucuugaacuuAcu    1    1   LT-3
......................uuccacagcuuucuugaacuuCcu    1    1   LCK-3
......................uuccacagcuuucuugaacuuucu    2    0   LCK-3
......................uuccacagcuuucuugaacuuuAu    2    1   LT-1
......................uuccacagcuuucuugaacuuuUu    49   1   JCK-3
......................uuccacagcuuucuugaacuuAcu    2    1   LT-2
......................uuccacagcuuucuugaacuuAcu    1    1   LT-1
......................uuccacagcuuucuugaacuuuAu    6    1   LT-3
......................uuccacagcuuucuugaacuuuUu    13   1   LT-3
......................uuccacagcuuucuugaacuuuAu    4    1   JT-1
......................uuccacagcuuucuugaacuuuAu    2    1   LT-2
......................uuccacagcuuucuugaacuuucA    1    1   LT-3
......................uuccacagcuuucuugaacuuuUu    19   1   LT-1
......................uuccacagcuuucuugaacuuuUu    47   1   JCK-1
......................uuccacagcuuucuugaacuAucu    1    1   JCK-3
......................uuccacagcuuucuugaacuuuUu    89   1   JCK-2
......................uuccacagcuuucuugaacuuucA    1    1   LT-1
......................uuccacagcuuucuugaacuuuUu    13   1   LCK-3
......................uuccacagcuuucuugaacuuuUu    15   1   LT-2
......................uuccacagcuuucuugaacuuucA    2    1   JCK-2
......................uuccacagcuuucuugaacuuucA    6    1   LT-2
......................uuccacagcuuucuugaacuuucu    3    0   LT-1
......................uuccacagcuuucuugaacuAucu    1    1   JCK-1
......................uuccacagcuuucuugaacuuuUuu   5    1   LT-2
......................uuccacagcuuucuugaacuuAcuu   3    1   JCK-2
......................uuccacagcuuucuugaacuuuUuu   9    1   JCK-1
......................uuccacagcuuucuugaacuuuUuu   12   1   JCK-2
......................uuccacagcuuucuugaacuuuAuu   8    1   JCK-2
......................uuccacagcuuucuugaacuuuAuu   5    1   LT-1
......................uuccacagcuuucuugaacuuuAuu   3    1   LT-3
......................uuccacagcuuucuugaacuuuUuu   1    1   LT-1
......................uuccacagcuuucuugaacuuuAuu   12   1   JCK-3
......................uuccacagcuuucuugaacuuuAuu   7    1   LCK-3
......................uuccacagcuuucuugaacuuuAuu   4    1   LT-2
......................uuccacagcuuucuugaacuuucuA   1    1   LT-2
......................uuccacagcuuucuugaacuuuUuu   2    1   LCK-3
......................uuccacagcuuucuugaacuuuGuu   1    1   LT-3
......................uuccacagcuuucuugaacuuuAuu   7    1   JCK-1
......................uuccacagcuuucuugaacuuucuC   1    1   LT-3
......................uuccacagcuuucuugaacuuuUuu   6    1   JCK-3
......................uuccacagcuuucuugaacuuuUuu   2    1   LT-3
......................uuccacagcuuucuugaacuuuUuuu  2    1   LCK-3
......................uuccacagcuuucuugaacuuuAuuu  3    1   LCK-3
......................uuccacagcuuucuugaacuuuAuuu  5    1   JCK-3
......................uuccacagcuuucuugaacuuucuuu  1    0   JCK-1
```

```
                      Mature                                                                            Star
uuugcaaguccuggucaugcuuuuccacagcuuucuugaacuuucuuucuuucuguuuagucucuuuuuuaagauugaacaccauugaaagcucaagaaagcugugggagaauauggcaaauuugggauuuuaauuuuuuuauuuuuguuuuuauuuuuuauuuuuccuucaacuacucuucagcugaugaugauguugcacacagaaagaaagaaagaaagaaagaaagagagaucucagaagcuaaau
......................uuccacagcuuucuugaacuuuAuuu..........................................................................................................................................................................................   3    1   JCK-2
......................uuccacagcuuucuugaacuuuAuuu..........................................................................................................................................................................................   3    1   JCK-1
.......................uccacagcuuucuugaac.................................................................................................................................................................................................   2    0   JT-2
.......................uccacagcuuucuugaac.................................................................................................................................................................................................   4    0   JT-3
.......................uccacagcuuucuugaac.................................................................................................................................................................................................   6    0   JT-1
.......................uccacaUcuuucuugaac.................................................................................................................................................................................................   1    1   LT-3
.......................uccacagcuuucuugaac.................................................................................................................................................................................................   2    0   JCK-2
.......................uccacagcuuucuugaac.................................................................................................................................................................................................   10   0   LT-2
.......................uccacagcuuucuugaac.................................................................................................................................................................................................   2    0   JCK-3
.......................uccacagcuuucuugaac.................................................................................................................................................................................................   11   0   LT-3
.......................Cccacagcuuucuugaac.................................................................................................................................................................................................   1    1   LT-1
.......................uccacagcuCucuugaac.................................................................................................................................................................................................   1    1   LT-3
.......................ucNacagcuuucuugaac.................................................................................................................................................................................................   1    1   LT-2
.......................uccacagcuuucuugaac.................................................................................................................................................................................................   13   0   LCK-3
.......................uccacagcuuucuugaac.................................................................................................................................................................................................   4    0   LT-1
.......................uccacagcuuucuugaacA................................................................................................................................................................................................   1    1   LT-1
.......................uccacagcuuucuugaaUu................................................................................................................................................................................................   1    1   LT-1
.......................uccacagcuuucuugaacu................................................................................................................................................................................................   29   0   LCK-2
.......................uccacagcuuucuugaacu................................................................................................................................................................................................   7    0   LT-1
.......................uccacagcuuucuugaacu................................................................................................................................................................................................   7    0   JCK-3
.......................uccacagcuuucuugaacu................................................................................................................................................................................................   10   0   JCK-2
.......................uccacagcuuucuugaaAu................................................................................................................................................................................................   3    1   LT-2
.......................uccacagcuuucuugaacu................................................................................................................................................................................................   53   0   LCK-1
.......................uccacagcuuucuugaacA................................................................................................................................................................................................   1    1   LT-3
.......................uccacagcuuucuugaacu................................................................................................................................................................................................   2    0   JCK-1
.......................uccacagcuuucuugaacu................................................................................................................................................................................................   2    0   LT-2
.......................uccacagcuuucuugaacu................................................................................................................................................................................................   12   0   LCK-3
.......................uccacagcuuucuugaacu................................................................................................................................................................................................   18   0   LT-3
.......................uccacagcuuucuugaaUuu...............................................................................................................................................................................................   1    1   LT-3
.......................uccacagcuuucuugaacuu...............................................................................................................................................................................................   9    0   LCK-3
.......................uccacagcuuucuugaacuu...............................................................................................................................................................................................   5    0   JCK-3
.......................uccacagcuuucuugaacuu...............................................................................................................................................................................................   2    0   LT-1
.......................uccacagcuuucuugaacuu...............................................................................................................................................................................................   2    0   LT-3
.......................uccacagcuuucuugaacuu...............................................................................................................................................................................................   6    0   JCK-2
.......................uccacagcuuucuugaacuu...............................................................................................................................................................................................   7    0   LT-2
.......................uccacagcuuucuugaacuu...............................................................................................................................................................................................   1    0   JCK-1
.......................uccacagcuuucuugaacuuu..............................................................................................................................................................................................   2    0   JCK-1
.......................uccacagcuuucuugaacuuu..............................................................................................................................................................................................   5    0   LT-2
.......................Cccacagcuuucuugaacuuu..............................................................................................................................................................................................   1    1   JCK-1
.......................uccacagcuuucuugaacAuu..............................................................................................................................................................................................   4    1   LT-2
.......................uccacagcuuucuugaacuuu..............................................................................................................................................................................................   1    0   JCK-2
.......................uccacagcuuucuugaacuuC..............................................................................................................................................................................................   1    1   LT-2
.......................uccacagcuuucuugaacuuu..............................................................................................................................................................................................   4    0   LT-3
.......................uccacagcuuucuugaacuuu..............................................................................................................................................................................................   4    0   LCK-3
.......................uccacagcuuucuugaacuuu..............................................................................................................................................................................................   2    0   LT-1
.......................uccacagcuuucuugaacuuuc.............................................................................................................................................................................................   1    0   JCK-2
.......................uccacagcuuucuugaacuuuc.............................................................................................................................................................................................   2    0   LT-1
.......................uccacagcuuucuugaacuuuA.............................................................................................................................................................................................   1    1   LT-2
.......................uccacagcuuucuugaacuuuU.............................................................................................................................................................................................   1    1   LT-2
.......................uccacagcuuucuugaacuuuc.............................................................................................................................................................................................   1    0   JCK-1
.......................uccacagcuuucuugaacuuuU.............................................................................................................................................................................................   1    1   JCK-1
.......................uccacagcuuucuugaacuuuA.............................................................................................................................................................................................   10   1   LCK-3
.......................uccacagcuuucuugaacuuuU.............................................................................................................................................................................................   6    1   LCK-3
.......................uccacagcuuucuugaacuuuU.............................................................................................................................................................................................   3    1   JCK-2
.......................uccacagcuuucuugaacuuuU.............................................................................................................................................................................................   2    1   JCK-3
.......................uccacagcuuucuugaacuuuU.............................................................................................................................................................................................   2    1   LT-3
.......................uccacagcuuucuugaacuuuU.............................................................................................................................................................................................   3    1   LT-1
.......................uccacagcuuucuugaacuuuUu............................................................................................................................................................................................   2    1   LCK-3
........................ccacagcuuucuugaacu................................................................................................................................................................................................   104  0   LT-1
........................ccacagcuuucuugaacA................................................................................................................................................................................................   1    1   JCK-1
........................ccCcagcuuucuugaacu................................................................................................................................................................................................   1    1   LT-2
........................ccacCgcuuucuugaacu................................................................................................................................................................................................   1    1   LT-3
........................ccacagcuuucuugaaUu................................................................................................................................................................................................   2    1   LCK-3
........................ccacagcuuucuugaacC................................................................................................................................................................................................   1    1   LT-3
........................Acacagcuuucuugaacu................................................................................................................................................................................................   1    1   LT-2
........................ccacagcCuucuugaacu................................................................................................................................................................................................   1    1   LT-3
........................ccacagcuuucuugaacu................................................................................................................................................................................................   301  0   LT-2
........................ccacagcuuucuugaaUu................................................................................................................................................................................................   5    1   LT-3
........................ccacagcuuuUuugaacu................................................................................................................................................................................................   4    1   LT-3
........................ccacagcuuucuugaacu................................................................................................................................................................................................   89   0   JCK-2
```

Mature **Star**

uuugcaaguccuggucaugcuuuuccacagcuuucuugaacuuucuuucuuucuguuuagucucuuuuuuaagauugaacaccauugaaagcucaagaaagcugugggagaauauggcaaauuugggauuuuaauuuuuuuauuuuuguuuuuauuuuuuauuuuuccuucaacuacucuucagcugaugaugauguugcacacagaaagaaagaaagaaagaaagaaagagagaucucagaagcuaaau

ccacagcuuucuugaacu	24	0	LCK-1
ccacagcuuucuugaacG	1	1	LT-3
ccacagcuuucuugaacC	2	1	JCK-1
ccacagcuuucuugaaUu	2	1	LT-1
ccacagcuuucuugaacu	20	0	JT-3
ccacagcuuucuugaacA	2	1	LT-2
ccacagcuuucuugaacu	342	0	LT-3
ccacagcuuucuugaCcu	2	1	LCK-3
ccacagcuuucuugCacu	2	1	LT-3
ccacagcuuucuugaacu	51	0	JCK-1
ccacagcuuucuugaacu	51	0	JT-1
ccacagcuuucuugaacu	36	0	LCK-2
ccacagcuuuAuugaacu	1	1	LT-3
ccacagcuuucuugaacu	69	0	JCK-3
ccacagcuuucuugaacu	318	0	LCK-3
ccacagcuuucuugaacu	14	0	JT-2
ccacagcuuucuugaCcu	1	1	LT-3
ccacagcuuucuugaacA	1	1	JCK-2
ccacagcuuucuugaCcu	1	1	JT-1
ccacagcuuucuugaacA	1	1	LT-3
ccacagcuuucuugaaUuu	4	1	JCK-3
ccacagcuuucuugCacuu	2	1	JCK-3
ccacagcuuucuugaacuC	5	1	LT-1
ccacagcuuucuugaCcuu	1	1	JT-2
ccacagcuuucuugaacuu	66	0	LT-1
ccacCgcuuucuugaacuu	2	1	LCK-3
ccCcagcuuucuugaacuu	1	1	LT-3
ccacCgcuuucuugaacuu	1	1	JT-1
ccaAagcuuucuugaacuu	1	1	JCK-1
ccacagcuuucuugaacuA	9	1	LT-3
ccacagcuuucuugaacuC	4	1	LT-3
ccacagcuuucuugaacuu	180	0	JCK-3
ccacagcuuucuugaaUuu	3	1	JCK-1
ccacagcuuucuugaacuu	34	0	JT-2
ccacagcuuucuugaaUuu	6	1	LCK-3
ccacagcuuucuugaacuC	2	1	JCK-1
ccacagcuuucuugaacuA	10	1	JT-1
ccacagcuuucuugaacAu	1	1	JCK-1
ccaNagcuuucuugaacuu	1	1	JCK-1
ccacagcuuucuugaacuA	19	1	LT-2
ccacagcuuucuugaacuC	4	1	JCK-3
ccacagcuuucuugaacuA	8	1	JCK-3
ccacagcuuucuugaaAuu	3	1	LT-3
ccacagcuuucuugaacuA	11	1	JCK-1
ccacagcuuucuugaaAuu	1	1	JCK-2
cNacagcuuucuugaacuu	1	1	JCK-2
ccacagcuuucuugaacuu	349	0	LCK-3
ccacagcuuucuugaCcuu	1	1	LT-2
ccacagcuuucuugaCcuu	1	1	LCK-3
cNacagcuuucuugaacuu	1	1	JCK-3
ccacagcuuucuugaaUuu	3	1	LT-1
ccacagcuuucuugaacuC	3	1	LT-2
ccacagcuuucuugaacuu	42	0	JT-3
ccacagcuuucuugaacuu	252	0	JCK-2
ccacagcuuucuugaacuu	246	0	LT-2
ccacagcuuucuugaacuA	41	1	LCK-3
cNacagcuuucuugaacuu	1	1	JT-1
ccacagcuuucuugaacuA	5	1	JT-3
ccacagcuuucuugaaUuu	9	1	LT-2
ccacagcuuucuugCacuu	1	1	JCK-2
ccacagcuuucuugaacuu	168	0	JCK-1
ccacagcuuucuugaacuu	68	0	JT-1
ccacagcuuucuugaacuA	17	1	JCK-2
ccacagcuuucuugaaUuu	5	1	JCK-2
ccacagcCuucuugaacuu	3	1	LCK-3
ccacagcuuucuugaacuA	6	1	LT-1
ccacagcuuucuugaacCu	1	1	LCK-3
ccacagcuuucNugaacuu	1	1	JCK-1
ccacagcuuucuugaaUuu	7	1	LT-3
ccacagcuuucuugaacuu	251	0	LT-3

```
                         Mature                                                       Star
uuugcaaguccuggucaugcuuuuccacagcuuucuugaacuuucuuucuuucuguuuagucucuuuuuuaagauugaacaccauugaaagcucaagaaagcugugggagaauauggcaaauuugggauuuuaauuuuuuuauuuuuguuuuuauuuuuuauuuuuccuucaacuacucuucagcugaugaugauguugcacacagaaagaaagaaagaaagaaagaaagagagaucucagaagcuaaau
........................ccacagcuuucuugaacuA................................................................................................................................................................................................................   3     1   JT-2
........................ccacagcuuucuugaacuC................................................................................................................................................................................................................   10    1   JT-1
........................ccacagcuuucuugaacuC................................................................................................................................................................................................................   12    1   JCK-2
........................ccacagcuuucuugCacuu................................................................................................................................................................................................................   2     1   LT-2
........................ccacCgcuuucuugaacuu................................................................................................................................................................................................................   1     1   JCK-2
........................ccacagcuuucuugaacuuC...............................................................................................................................................................................................................   20    1   LT-3
........................ccacCgcuuucuugaacuuu...............................................................................................................................................................................................................   3     1   JCK-3
........................ccacagcuuucuugaacuuA...............................................................................................................................................................................................................   8     1   JT-3
........................ccacagcuuucuugaacuuG...............................................................................................................................................................................................................   1     1   JCK-3
........................ccacagcuuucuugaacuuG...............................................................................................................................................................................................................   1     1   LT-1
........................ccNcagcuuucuugaacuuu...............................................................................................................................................................................................................   1     1   LCK-1
........................ccacagcuuucuuAaacuuu...............................................................................................................................................................................................................   1     1   LT-2
........................ccacagcuuucuugaacuuu...............................................................................................................................................................................................................   349   0   JCK-3
........................ccacagcuuucuugaacuuu...............................................................................................................................................................................................................   77    0   LCK-1
........................ccacagcuuucuugaacuuG...............................................................................................................................................................................................................   7     1   JCK-1
........................cNacagcuuucuugaacuuu...............................................................................................................................................................................................................   1     1   JCK-1
........................ccacagcuuucuugaacuuu...............................................................................................................................................................................................................   334   0   JCK-1
........................ccacagcuuucuugaacuuA...............................................................................................................................................................................................................   51    1   LT-2
........................ccacagcuuucuugaacuuA...............................................................................................................................................................................................................   48    1   LT-3
........................ccacagcuuucuugaacuuC...............................................................................................................................................................................................................   24    1   JCK-2
........................Ucacagcuuucuugaacuuu...............................................................................................................................................................................................................   4     1   JCK-2
........................ccacagcuuucuugaacuuu...............................................................................................................................................................................................................   169   0   LT-1
........................ccacagcuuucuugaaUuuu...............................................................................................................................................................................................................   3     1   JCK-3
........................ccacagcuuucuugaacuuC...............................................................................................................................................................................................................   15    1   JCK-1
........................ccacagcuuucuugaacuAu...............................................................................................................................................................................................................   6     1   JCK-1
........................ccacCgcuuucuugaacuuu...............................................................................................................................................................................................................   2     1   LT-2
........................ccacagcuuucuugaaUuuu...............................................................................................................................................................................................................   27    1   LCK-1
........................ccacagcuuucuugaaUuuu...............................................................................................................................................................................................................   4     1   JCK-1
........................ccacagcuuucuugaacuCu...............................................................................................................................................................................................................   4     1   LT-1
........................Ucacagcuuucuugaacuuu...............................................................................................................................................................................................................   1     1   JCK-1
........................ccacagcuuucuugaacAuu...............................................................................................................................................................................................................   5     1   LCK-3
........................ccacagcuuucuugaacuuu...............................................................................................................................................................................................................   43    0   JT-1
........................ccacagGuuucuugaacuuu...............................................................................................................................................................................................................   1     1   JT-3
........................ccacagcuuucuugaacuAu...............................................................................................................................................................................................................   5     1   JCK-3
........................ccacagcuuucuugaacuuA...............................................................................................................................................................................................................   10    1   LT-1
........................ccacagcuuucuugaacuCu...............................................................................................................................................................................................................   7     1   JCK-3
........................ccacagcuuucuugNacuuu...............................................................................................................................................................................................................   1     1   LCK-1
........................ccacagcuuucuugaacuuC...............................................................................................................................................................................................................   18    1   JCK-3
........................ccacagcuuucuugaacuuu...............................................................................................................................................................................................................   485   0   LT-2
........................ccacagcuuucuugaGcuuu...............................................................................................................................................................................................................   1     1   LCK-2
........................ccacagcuuucuugaacuuA...............................................................................................................................................................................................................   69    1   JCK-2
........................ccacagcuuucuugaacuuu...............................................................................................................................................................................................................   31    0   JT-3
........................ccacagcuuucuugaacuuu...............................................................................................................................................................................................................   495   0   JCK-2
........................ccacagcuuucuugaacuCu...............................................................................................................................................................................................................   8     1   LT-3
........................ccacagcuuucuugaacGuu...............................................................................................................................................................................................................   1     1   JCK-1
........................ccacagcuuucuugaacAuu...............................................................................................................................................................................................................   3     1   LT-2
........................ccacagcuuucuugaacuuC...............................................................................................................................................................................................................   5     1   LCK-3
........................ccacagcuuucuugaacuuC...............................................................................................................................................................................................................   14    1   LT-2
........................ccaAagcuuucuugaacuuu...............................................................................................................................................................................................................   1     1   LCK-1
........................ccacagcuuucuugaacuuG...............................................................................................................................................................................................................   2     1   JCK-2
........................ccacCgcuuucuugaacuuu...............................................................................................................................................................................................................   1     1   JCK-1
........................ccacagcuuucuugaCcuuu...............................................................................................................................................................................................................   1     1   JCK-3
........................ccacagcuuucuugaacuAu...............................................................................................................................................................................................................   9     1   JCK-2
........................ccacagcuuucuugaacuuu...............................................................................................................................................................................................................   21    0   JT-2
........................ccacaAcuuucuugaacuuu...............................................................................................................................................................................................................   1     1   JCK-3
........................ccacagcuuucuugaacuAu...............................................................................................................................................................................................................   16    1   LT-2
........................ccacagcuuucuugCacuuu...............................................................................................................................................................................................................   1     1   LCK-3
........................ccacagcuuucuugaaUuuu...............................................................................................................................................................................................................   7     1   LT-3
........................ccacagcuuucuugaacAuu...............................................................................................................................................................................................................   1     1   JCK-2
........................ccacagcuuucuugaacuuC...............................................................................................................................................................................................................   4     1   LT-1
........................ccacagcuuucuugaaUuuu...............................................................................................................................................................................................................   13    1   LCK-3
........................ccacagcuuucuugaacuuA...............................................................................................................................................................................................................   53    1   JCK-3
........................ccacagcuuCcuugaacuuu...............................................................................................................................................................................................................   1     1   LT-3
........................ccacagcuuucuugaacuCu...............................................................................................................................................................................................................   17    1   JCK-2
........................ccacagcuuucuugaacuuA...............................................................................................................................................................................................................   41    1   JCK-1
........................ccacagcuuucuugaacuuu...............................................................................................................................................................................................................   71    0   LCK-2
........................ccacagcuuucuugaacuCu...............................................................................................................................................................................................................   4     1   LT-2
........................ccacagcuuucuugaacuuA...............................................................................................................................................................................................................   40    1   LCK-3
........................ccacagcuuucuugaacAuu...............................................................................................................................................................................................................   2     1   JCK-1
........................ccacagcuuucuugaacAuu...............................................................................................................................................................................................................   2     1   LT-1
```

Mature **Star**

```
uuugcaaguccuggucaugcuuuuccacagcuuucuugaacuuucuuucuuucuguuuagucucuuuuuuaagauugaacaccauugaaagcucaagaaagcugugggagaauauggcaaauuugggauuuuaauuuuuuuauuuuuguuuuuauuuuuuauuuuuccuucaacuacucuucagcugaugaugauguugcacacagaaagaaagaaagaaagaaagaaagagagaucucagaagcuaaau
........................ccacagcCuucuugaacuuu..............................................................................................................................................................................................   1     1   JCK-3
........................ccacCgcuuucuugaacuuu..............................................................................................................................................................................................   1     1   JT-1
........................ccacagcuuucuugCacuuu..............................................................................................................................................................................................   1     1   LT-2
........................ccacagcuuucuugaacuuu..............................................................................................................................................................................................   439   0   LT-3
........................ccacagcuuucuugaacuuG..............................................................................................................................................................................................   1     1   LT-3
........................ccacagcuuucuugaacAuu..............................................................................................................................................................................................   2     1   LT-3
........................ccacagcuuucuugaacuuu..............................................................................................................................................................................................   506   0   LCK-3
........................ccacagcuuucuugaacuCu..............................................................................................................................................................................................   4     1   JCK-1
........................ccacaNcuuucuugaacuuu..............................................................................................................................................................................................   1     1   LCK-1
........................ccacagcuuucuugCacuuu..............................................................................................................................................................................................   1     1   JCK-2
........................ccacagcuuucuugaaAuuu..............................................................................................................................................................................................   5     1   LCK-3
........................ccacagcuuucuugaacuAu..............................................................................................................................................................................................   9     1   LT-3
........................ccacagcuuucuugaacuuA..............................................................................................................................................................................................   11    1   JT-2
........................ccacagcuuucuugaacuAu..............................................................................................................................................................................................   9     1   LCK-3
........................ccacagcuuucuugaaUuuu..............................................................................................................................................................................................   18    1   LCK-2
........................ccacagcuuucuugaacuuuU.............................................................................................................................................................................................   153   1   JCK-3
........................ccacagcuuucuugaacuuuc.............................................................................................................................................................................................   9     0   JCK-3
........................ccacagcuuucuugaacuuAc.............................................................................................................................................................................................   1     1   JCK-2
........................ccacagcuuucuugaacuuuA.............................................................................................................................................................................................   146   1   LT-2
........................ccacagcuuucuugaacuAuc.............................................................................................................................................................................................   1     1   LT-3
........................ccacagcuuucuugaacuuCc.............................................................................................................................................................................................   1     1   JCK-1
........................ccacagcuuucuugaacuuuc.............................................................................................................................................................................................   8     0   JCK-2
........................ccacagcuuucuugaacuuuA.............................................................................................................................................................................................   178   1   JCK-2
........................ccacagcuuucuugaacuuuU.............................................................................................................................................................................................   64    1   LCK-2
........................ccacagcuuucuugaacuuuG.............................................................................................................................................................................................   6     1   LT-3
........................ccacagcuuucuugaacuuuA.............................................................................................................................................................................................   33    1   LT-1
........................ccacagcuuucuugaacuuuU.............................................................................................................................................................................................   246   1   LT-2
........................ccacagcuuucuugaacuuuA.............................................................................................................................................................................................   90    1   JCK-3
........................ccacagcuuucuugaacuuCc.............................................................................................................................................................................................   1     1   LT-3
........................ccacagcuuucuugaacuuuU.............................................................................................................................................................................................   42    1   JT-1
........................ccacagcuuucuugaacuuuG.............................................................................................................................................................................................   8     1   JCK-2
........................ccacagcuuucuugaaUuuuc.............................................................................................................................................................................................   1     1   LT-2
........................ccacagcuuucuugaacuuAc.............................................................................................................................................................................................   5     1   LCK-3
........................ccacagcuuucuugaacuuuU.............................................................................................................................................................................................   13    1   JT-3
........................ccacagcuuucuugaacuuuG.............................................................................................................................................................................................   9     1   JCK-1
........................ccacagcuuucuugaacuuuU.............................................................................................................................................................................................   52    1   LCK-1
........................ccacagcuuucuugaacuuuA.............................................................................................................................................................................................   109   1   LCK-3
........................ccacagcuuucuugaacuuuU.............................................................................................................................................................................................   184   1   JCK-2
........................ccacagcuuucuugaacuuuU.............................................................................................................................................................................................   349   1   LCK-3
........................ccacagcuuucuugaacuuAc.............................................................................................................................................................................................   3     1   LT-3
........................ccacagcuuucuugaacuuuG.............................................................................................................................................................................................   17    1   LCK-3
........................ccacagcuuucuugaacuuuG.............................................................................................................................................................................................   6     1   LT-1
........................ccacagcuuucuugaacuuuc.............................................................................................................................................................................................   16    0   LT-2
........................ccacagcuuucuugaacuuuG.............................................................................................................................................................................................   10    1   JCK-3
........................ccacagcuuucuugaacuuuA.............................................................................................................................................................................................   82    1   LT-3
........................ccacagcuuucuugaacuuuc.............................................................................................................................................................................................   29    0   LCK-3
........................ccacagcuuucuugaacuAuc.............................................................................................................................................................................................   7     1   LCK-3
........................ccacagcuuucuugaacuuuA.............................................................................................................................................................................................   150   1   JCK-1
........................ccacagcuuucuugaacuuuA.............................................................................................................................................................................................   7     1   JT-1
........................ccacagcuuucuugaacuuuc.............................................................................................................................................................................................   30    0   LCK-2
........................ccacagcuuucuugaacuuuU.............................................................................................................................................................................................   223   1   LT-3
........................ccacagcuuucuugaacuuuG.............................................................................................................................................................................................   14    1   LT-2
........................ccacagcuuucuugaacuuAc.............................................................................................................................................................................................   1     1   LT-2
........................ccacagcuuucuugaacuuuU.............................................................................................................................................................................................   138   1   JCK-1
........................ccacagcuuucuugaacuuuc.............................................................................................................................................................................................   8     0   LT-3
........................ccacagcuuucuugaacuuCc.............................................................................................................................................................................................   1     1   LCK-3
........................ccacagcuuucuugaCcuuuc.............................................................................................................................................................................................   1     1   LT-1
........................ccacagcuuucuugaacuuuU.............................................................................................................................................................................................   87    1   LT-1
........................ccacagcuuucuugaacuuuc.............................................................................................................................................................................................   4     0   JCK-1
........................ccacagcuuucuugaacuuuc.............................................................................................................................................................................................   3     0   LT-1
........................ccacagcuuucuugaacuuuc.............................................................................................................................................................................................   27    0   LCK-1
........................ccacagcuuucuugaacuuuU.............................................................................................................................................................................................   2     1   JT-2
........................ccacagcuuucuugaacuuucu............................................................................................................................................................................................   3     0   LT-3
........................ccacagcuuucuugaacuuuAu............................................................................................................................................................................................   1     1   JT-3
........................ccacagcuuucuugaacuuuUu............................................................................................................................................................................................   4     1   JT-3
........................ccacagcuuucuugaacuuuAu............................................................................................................................................................................................   16    1   JCK-3
........................ccacagcuuucuugaacuuucA............................................................................................................................................................................................   3     1   JCK-3
........................ccacagcuuucuugaacuuucu............................................................................................................................................................................................   1     0   LT-1
........................ccacagcuuucuugaacuuucA............................................................................................................................................................................................   5     1   JCK-2
........................ccacagcuuucuugaacuuucA............................................................................................................................................................................................   1     1   JCK-1
```

Mature Star

uuugcaaguccuggucaugcuuuuccacagcuuucuugaacuuucuuucuuucuguuuagucucuuuuuuaagauugaacaccauugaaagcucaagaaagcugugggagaauauggcaaauuugggauuuuaauuuuuuuauuuuuguuuuuauuuuuuauuuuuccuucaacuacucuucagcugaugaugauguugcacacagaaagaaagaaagaaagaaagaaagagagaucucagaagcuaaau			
........................ccacagcuuucuugaacuuucu..	4	0	JCK-2
........................ccacagcuuucuugaacuuuUu..	25	1	LT-3
........................ccacagcuuucuugaacuuAcu..	4	1	JCK-3
........................ccacagcuuucuugaacuuuAu..	4	1	LT-1
........................ccacagcuuucuugaacuuAcu..	1	1	JCK-1
........................ccacagcuuucuugaacuuAcu..	3	1	LT-2
........................ccacagcuuucuugaacuuuUu..	7	1	LT-1
........................ccacagcuuucuugaacuuAcu..	1	1	LT-1
........................ccacagcuuucuugaacuuucA..	4	1	LT-2
........................ccacagcuuucuugaacuuuUu..	29	1	JCK-1
........................ccacagcuuucuugaacuuuGu..	5	1	LCK-3
........................ccacagcuuucuugaacuuuUu..	39	1	LT-2
........................ccacagcuuucuugaacuuuAu..	19	1	JCK-1
........................ccacagcuuucuugaacuAucu..	2	1	LCK-3
........................ccacagcuuucuugaacuuucA..	3	1	LT-3
........................ccacagcuuucuugaacuuucu..	4	0	JCK-3
........................ccacagcuuucuugaacuuAcu..	23	1	LCK-3
........................ccacagcuuucuugaacuuAcu..	2	1	LT-3
........................ccacagcuuucuugaacuuuAu..	13	1	LT-3
........................ccacagcuuucuugaacuuuUu..	38	1	JCK-3
........................ccacagcuuucuugaacuuuAu..	16	1	LT-2
........................ccacagcuuucuugaacuuuAu..	23	1	LCK-3
........................ccacagcuuucuugaacuuuUu..	22	1	LCK-3
........................ccacagcuuucuugaacuuucG..	1	1	JCK-3
........................ccacagcuuucuugaacuuuUu..	2	1	JT-2
........................ccacagcuuucuugaacuuuGu..	3	1	LT-1
........................ccacagcuuucuugaacuuuAu..	23	1	JCK-2
........................ccacagcuuucuugaacuuucu..	1	0	JCK-1
........................ccacagcuuucuugaacuuucG..	5	1	JCK-2
........................ccacagcuuucuugaacuuAcu..	4	1	JCK-2
........................ccacagcuuucuugaacuuuUu..	54	1	JCK-2
........................ccacagcuuucuugaacuuucC..	2	1	LT-3
........................ccacagcuuucuugaacuuuAuu...	21	1	JCK-2
........................ccacagcuuucuugaacuuucCu...	1	1	JCK-2
........................ccacagcuuucuugaacuuuUuu...	11	1	JCK-2
........................ccacagcuuucuugaacuuucuA...	1	1	LT-2
........................ccacagcuuucuugaacuuuGuu...	1	1	JCK-2
........................ccacagcuuucuugaacuuuAuu...	13	1	LT-2
........................ccacagcuuucuugaacuuuAuu...	25	1	LCK-3
........................ccacagcuuucuugaacuuuUuu...	4	1	JCK-1
........................ccacagcuuucuugaacuuuAuu...	4	1	JT-3
........................ccacagcuuucuugaacuuuUuu...	5	1	LT-1
........................ccacagcuuucuugaacuuuUuu...	14	1	LCK-3
........................ccacagcuuucuugaacuuuUuu...	4	1	LT-3
........................ccacagcuuucuugaacuuuAuu...	3	1	LT-1
........................ccacagcuuucuugaacuuucAu...	1	1	JCK-3
........................ccacagcuuucuugaacuuuAuu...	13	1	JCK-3
........................ccacagcuuucuugaacuuucAu...	1	1	JCK-2
........................ccacagcuuucuugaacuuuUuu...	9	1	JCK-3
........................ccacagcuuucuugaacuuucuA...	2	1	LCK-3
........................ccacagcuuucuugaacuuuAuu...	14	1	JCK-1
........................ccacagcuuucuugaacuuuUuu...	5	1	LT-2
........................ccacagcuuucuugaacuuucuu...	1	0	JCK-3
........................ccacagcuuucuugaacuuuAuu...	1	1	JT-2
........................ccacagcuuucuugaacuuuAuu...	4	1	LT-3
........................ccacagcuuucuugaacuuuAuuu..	2	1	JCK-2
........................ccacagcuuucuugaacuuuAuuu..	1	1	LT-1
........................ccacagcuuucuugaacuuuUuuu..	3	1	LT-3
........................ccacagcuuucuugaacuuuUuuu..	3	1	JCK-3
........................ccacagcuuucuugaacuuuUuuu..	2	1	JCK-1
........................ccacagcuuucuugaacuuucAuu..	1	1	JCK-2
........................ccacagcuuucuugaacuuuUuuu..	2	1	JCK-2
........................ccacagcuuucuugaacuuuAuuu..	1	1	JCK-3
........................ccacagcuuucuugaacuuuAuuu..	3	1	JCK-1
........................ccacagcuuucuugaacuuuAuuu..	1	1	LT-3
........................ccacagcuuucuugaacuuuAuuu..	1	1	LT-2
........................ccacagcuuucuugaacuuucuCu..	1	1	JCK-2
........................ccacagcuuucuugaacuuuUuuu..	1	1	LT-2
.........................cacagcuuucuugaacuu...	3	0	LT-2
.........................cCcagcuuucuugaacuu...	1	1	LCK-3

```
                      Mature                                                              Star
uuugcaaguccuggucaugcuuuuccacagcuuucuugaacuuucuuucuuucuguuuagucucuuuuuuaagauugaacaccauugaaagcucaagaaagcugugggagaauauggcaaauuugggauuuuaauuuuuuuauuuuuguuuuuauuuuuuauuuuuccuucaacuacucuucagcugaugaugauguugcacacagaaagaaagaaagaaagaaagaaagagagaucucagaagcuaaau
........................cacagcuuucuugaacuu.........................................................................................................................................................................................................   3   0   JCK-3
........................cacagcuuucuugaacuu.........................................................................................................................................................................................................   5   0   LCK-3
........................cacagcuuucuugaacuu.........................................................................................................................................................................................................   1   0   LT-3
........................cacagcuuucuugaacuuu........................................................................................................................................................................................................   8   0   JCK-2
........................cacagcuuucuugaacuuu........................................................................................................................................................................................................   4   0   LT-2
........................cacagcuuucuugaacuuu........................................................................................................................................................................................................   2   0   JCK-3
........................cacagcuuucuugaacuAu........................................................................................................................................................................................................   2   1   LT-2
........................cacagcuuucuugaacuuu........................................................................................................................................................................................................   1   0   JT-2
........................cacagcuuucuugaacuuu........................................................................................................................................................................................................   1   0   JCK-1
........................cacagcuuucuugaacuuA........................................................................................................................................................................................................   1   1   JCK-1
........................cacagcuuucuugaacuuu........................................................................................................................................................................................................   10  0   JT-3
........................cacagcuuucuugaacuuu........................................................................................................................................................................................................   6   0   LT-3
........................cacagcuuucuugaacuuA........................................................................................................................................................................................................   1   1   LT-3
........................cacagcuuucuugaacuuu........................................................................................................................................................................................................   2   0   LT-1
........................cacagcuuucuugaacuuA........................................................................................................................................................................................................   2   1   JCK-3
........................cacagcuuucuugaacuuuU.......................................................................................................................................................................................................   5   1   JCK-3
........................cacagcuuucuugaacuuuU.......................................................................................................................................................................................................   1   1   LT-1
........................cacagcuuucuugaacuuuU.......................................................................................................................................................................................................   3   1   JCK-1
........................cacagcuuucuugaacuuuU.......................................................................................................................................................................................................   5   1   JCK-2
........................cacagcuuucuugaacuuuU.......................................................................................................................................................................................................   2   1   LT-2
........................cacagcuuucuugaacuuuU.......................................................................................................................................................................................................   2   1   LT-3
........................cacagcuuucuugaacuuuA.......................................................................................................................................................................................................   4   1   LCK-3
........................cacagcuuucuugaacuuuU.......................................................................................................................................................................................................   5   1   LCK-3
........................cacagcuuucuugaacuuuA.......................................................................................................................................................................................................   1   1   JCK-3
........................cacagcuuucuugaacuuuA.......................................................................................................................................................................................................   1   1   LT-3
........................cacagcuuucuugaacuuucu......................................................................................................................................................................................................   3   0   LT-1
........................cacagcuuucuugaacuuucu......................................................................................................................................................................................................   9   0   JCK-3
........................cacagcuuucuugaacuuucu......................................................................................................................................................................................................   2   0   JCK-2
........................cacagcuuucuugaacuuucu......................................................................................................................................................................................................   2   0   JCK-1
........................cacagcuuucuugaacuuuUuu.....................................................................................................................................................................................................   2   1   LT-3
........................cacagcuuucuugaacuuuAuu.....................................................................................................................................................................................................   3   1   JCK-1
........................cacagcuuucuugaacuuuUuu.....................................................................................................................................................................................................   1   1   LT-1
........................cacagcuuucuugaacuuuAuu.....................................................................................................................................................................................................   2   1   JCK-3
........................cacagcuuucuugaacuuuAuu.....................................................................................................................................................................................................   8   1   JCK-2
........................cacagcuuucuugaacuuAcuuu....................................................................................................................................................................................................   1   1   LT-1
.........................acagcuuucuugaacuuu........................................................................................................................................................................................................   1   0   LT-2
.........................acagcuuucuugaaUuuu........................................................................................................................................................................................................   6   1   LCK-3
.........................acagcuuucuugaacuuA........................................................................................................................................................................................................   2   1   LT-2
.........................acagcuuucuugaacuuuU.......................................................................................................................................................................................................   3   1   LT-2
.........................acagcuuucuugaacuuuA.......................................................................................................................................................................................................   1   1   LT-1
.........................acagcuuucuugaacuuuU.......................................................................................................................................................................................................   4   1   LCK-3
.........................acagcuuucuugaacuuuU.......................................................................................................................................................................................................   1   1   LT-3
.........................acagcuuucuugaacuuuUu......................................................................................................................................................................................................   3   1   LT-3
.........................................uuucuGucuuucuguuua.........................................................................................................................................................................................   1   1   LT-2
..........................................................................................gcucaagaaagcuguggg..........................................................................................................................................   1   0   JCK-2
..........................................................................................gcucaagaaagcugugga..........................................................................................................................................   1   0   LT-3
..........................................................................................gcucaagaaagcugugggag........................................................................................................................................   7   0   JT-3
..........................................................................................gcucaagaaagcugugggag........................................................................................................................................   2   0   JCK-1
..........................................................................................gcucaCgaaagcugugggag........................................................................................................................................   1   1   JT-3
..........................................................................................gcucaagaaagcugugggag........................................................................................................................................   2   0   JCK-3
..........................................................................................gcucaagaaagcugugggag........................................................................................................................................   3   0   JT-2
..........................................................................................gcucaagaaagcugugggaga.......................................................................................................................................   13  0   JCK-1
..........................................................................................gcucaagaaagcugugggaga.......................................................................................................................................   1   0   LT-1
..........................................................................................gcucaagaaagcugugggaga.......................................................................................................................................   17  0   JCK-3
..........................................................................................Acucaagaaagcugugggaga.......................................................................................................................................   1   1   JCK-1
..........................................................................................gcucaagaaagcugugggaga.......................................................................................................................................   7   0   LCK-3
..........................................................................................gcucaagaaagcugugggaga.......................................................................................................................................   5   0   LT-3
..........................................................................................gcucaagaaagcugugggaga.......................................................................................................................................   17  0   JCK-2
...........................................................................................cucaagaaagcugugga..........................................................................................................................................   2   0   JCK-3
...........................................................................................cucaagaaagcugugga..........................................................................................................................................   1   0   JCK-2
...........................................................................................cucaagaaagcugugggag........................................................................................................................................   4   0   JT-3
...........................................................................................cucaagaaagcugugggag........................................................................................................................................   2   0   JCK-1
...........................................................................................cucaagaaagcugugggag........................................................................................................................................   6   0   JT-2
...........................................................................................cucaagaaagcugugggag........................................................................................................................................   1   0   JCK-3
...........................................................................................cucaagaaagcugugggag........................................................................................................................................   4   0   JCK-2
...........................................................................................cucaagaaagcugugggag........................................................................................................................................   1   0   LT-3
...........................................................................................cucaagaaagcugugggaga.......................................................................................................................................   112 0   JCK-3
...........................................................................................cucaagaaagcugugggaga.......................................................................................................................................   86  0   JCK-1
...........................................................................................cucaagaaagcugugggagC.......................................................................................................................................   2   1   JCK-3
...........................................................................................Uucaagaaagcugugggaga.......................................................................................................................................   1   1   JCK-2
```

Mature **Star**

uuugcaaguccuggucaugcuu**uuccacagcuuucuugaacu**uucuuucuuucuguuuagucucuuuuuuaagauugaacaccauugaaag**cucaagaaagcugugggaga**auauggcaaauuugggauuuuaauuuuuuuauuuuuguuuuuauuuuuuauuuuuccuucaacuacucuucagcugaugaugauguugcacacagaaagaaagaaagaaagaaagaaagagagaucucagaagcuaaau

cucaagaaagcugugggaga	19	0	LT-1
cucaaUaaagcugugggaga	3	1	JCK-3
cucaagaaagAugugggaga	1	1	JCK-2
cNcaagaaagcugugggaga	2	1	JCK-3
cucaagaaagcugugggaga	6	0	JT-2
cucaCgaaagcugugggaga	1	1	JCK-2
cucaagaaagcugugggaga	8	0	JT-3
cucaagaaagcugugggaga	23	0	LT-3
cucaagaaagcugugggaga	21	0	LT-2
cucaagaaagcugugggaga	111	0	JCK-2
cucaagaaagcugugggagC	2	1	JCK-2
cucaagaaaAcugugggaga	1	1	LT-2
cucaagaaagcugugggagaU	1	1	JCK-3
cucaagaaagcugugggagaC	2	1	JCK-2
cucaagaaagcugugggagaC	1	1	JCK-1
cucaagaaagcugugggagaUu	1	1	LT-3
ucaagaaagcugugggaga	1	0	JCK-1
ucaagaaagcugugggaga	3	0	JCK-2
ucaagaaagcugugggaga	1	0	JCK-3
aagaaagcugugggagaauauggc	2	0	JT-3
aagaaagcugugggagaauauggc	1	0	LT-3
aagaaagcugugggagaauauggc	10	0	JCK-3
aagaaagcugugggagaauauggc	1	0	LT-1
aagaaagcugugggagaauauggc	4	0	JT-2
aagaaagcugugggagaauauggc	10	0	JCK-2
aagaaagcugugggagaauauggc	4	0	JCK-1
aaagcugugggagaauauggc	1	0	JCK-3
aaagcugugggagaauauggc	2	0	JCK-2
aagcugugggagaauauggc	3	0	LT-1
aagcugugggagaauauggc	1	0	JCK-3
aagcugugggagaauauggc	1	0	JCK-2
aagcugugggagaauauggc	3	0	LT-2
aagcugugggagaauauggca	6	0	JCK-2
aagcugugggagaauauggca	5	0	JT-3
aagcugugggagaauauggca	8	0	JT-2
aagcugugggagaauauggca	3	0	LT-3
agcugugggagaauauggc	3	0	JCK-2
ugugggagaauauggcaaauu	1	0	JCK-3
ugugggagaauauggcaaauu	1	0	JCK-2
